# 8-OxoG-Dependent Regulation of Global Protein Responses Leads to Mutagenesis and Stress Survival in *Bacillus subtilis*

**DOI:** 10.3390/antiox13030332

**Published:** 2024-03-08

**Authors:** Lissett E. Martínez, Gerardo Gómez, Norma Ramírez, Bernardo Franco, Eduardo A. Robleto, Mario Pedraza-Reyes

**Affiliations:** 1 Department of Biology, Division of Natural and Exact Sciences, University of Guanajuato, Guanajuato 36050, Mexico; le.martinezmagana@ugto.mx (L.E.M.); g.gomezromo@gmail.com (G.G.); nrramirez@ugto.mx (N.R.); bfranco@ugto.mx (B.F.); 2 School of Life Sciences, University of Nevada, Las Vegas, NV 89557, USA; eduardo.robleto@unlv.edu

**Keywords:** *Bacillus subtilis*, 8-oxoG, GO system, mutagenesis, oxidative stress

## Abstract

The guanine oxidized (GO) system of *Bacillus subtilis*, composed of the YtkD (MutT), MutM and MutY proteins, counteracts the cytotoxic and genotoxic effects of the oxidized nucleobase 8-OxoG. Here, we report that in growing *B. subtilis* cells, the genetic inactivation of GO system potentiated mutagenesis (HPM), and subsequent hyperresistance, contributes to the damaging effects of hydrogen peroxide (H_2_O_2_) (HPHR). The mechanism(s) that connect the accumulation of the mutagenic lesion 8-OxoG with the ability of *B. subtilis* to evolve and survive the noxious effects of oxidative stress were dissected. Genetic and biochemical evidence indicated that the synthesis of KatA was exacerbated, in a PerR-independent manner, and the transcriptional coupling repair factor, Mfd, contributed to HPHR and HPM of the ΔGO strain. Moreover, these phenotypes are associated with wider pleiotropic effects, as revealed by a global proteome analysis. The inactivation of the GO system results in the upregulated production of KatA, and it reprograms the synthesis of the proteins involved in distinct types of cellular stress; this has a direct impact on (*i*) cysteine catabolism, (*ii*) the synthesis of iron–sulfur clusters, (*iii*) the reorganization of cell wall architecture, (*iv*) the activation of AhpC/AhpF-independent organic peroxide resistance, and (*v*) increased resistance to transcription-acting antibiotics. Therefore, to contend with the cytotoxic and genotoxic effects derived from the accumulation of 8-OxoG, *B. subtilis* activates the synthesis of proteins belonging to transcriptional regulons that respond to a wide, diverse range of cell stressors.

## 1. Introduction

Microorganisms have evolved mechanisms to counteract the noxious effects promoted by oxidative stress [1,2,3]. Reactive oxygen species (ROS), including the superoxide anion (O_2_^•−^) and the hydroxyl radical (HO^•^), are byproducts generated during aerobic bacterial growth. They have the potential to modify the chemical structure of proteins, lipids, and nucleic acids [4,5]. Alterations to transition metals’ homeostasis also contributes to oxidative stress in bacteria, as hydrogen peroxide (H_2_O_2_) can elicit the autocatalytic production of HO^•^ from reduced Fe(II) and Cu(I) via redox cycling with cellular reductants [6,7,8,9,10]. Therefore, the ability of microorganisms to rapidly detect and counteract ROS is critical for their survival. ROS-induced damage to cellular components activates specific and general stress responses in bacteria [5,11]. In some cases, oxygen and nitrogen radicals which act as secondary messengers (signals) are responsible for activating stress regulons that counteract and/or to adapt to the deleterious effects of oxidative stress [11]. In *Bacillus subtilis*, the transcriptional reprogramming that occurs in response to peroxide stress is carried out by the general stress response sigma factor, (σ^B^), and two transcriptional repressors, OhrR and PerR [12,13]. 

During the transition from exponential growth to the stationary phase of growth, *B. subtilis* activates the synthesis of σ^B^; this alternative sigma factor regulates the transcription of ~200 genes encoding general stress proteins (GSP). Examples of ROS-protecting proteins/enzymes under σ^B^ regulation include KatB, KatX, Dps, OhrB, and TxrA [13,14]. In *B. subtilis*, PerR controls the expression of genes required for ROS and iron detoxification, including, *katA*, *ahpCF*, *hemAXCDL*, *mrgA* and *pfeT*; notably, PerR also controls its own synthesis and that from the Fur protein [15]. OhrR specifically senses organic peroxides and is a repressor of a gene encoding a thiol peroxidase (OhrA) [16]. These regulons have been elucidated by experiments examining how cells respond to exposure to oxidants; these include hydrogen peroxide, paraquat, organic peroxides, and diamide [17].

During ROS attacks on DNA, a wide variety of oxidized bases are produced; however, guanine is more prone to oxidation due to its low redox potential [18,19,20]. The resulting 8-hydroxyguanine (8-OxoG) lesion, which can also be incorporated into DNA with the 8-Oxo-dGTP precursor, impacts the cells via its mutagenic and cytotoxic properties across the three domains of life [21,22,23]. In bacteria, the mutagenic effect of this lesion is counteracted by the proteins MutT, MutM, and MutY; altogether, this is called the guanine oxidized (GO) system [24]. This prevention/repair system constitutes an error-avoidance pathway that avoids the mutagenic effects of 8-OxoG, which can pair not only with cytosine (C), but also with adenine (A) during replication, resulting in a G:C to T:A transversion mutation [24,25,26].

In *B. subtilis*, the GO system is composed of YtkD (MutTA) encoding a nucleotide diphosphohydrolase (NUDIX) protein that transforms 8-oxo-dGTP into 8-oxo-dGMP, which can no longer function as a substrate for DNA polymerase, thus preventing its incorporation into DNA [27,28]. Two DNA glycosylases, MutM and MutY, complete the GO system in this bacterium. MutM removes 8-OxoG paired with cytosine from DNA, and MutY removes any adenine that is erroneously incorporated opposite to 8-OxoG [29,30].

Several lines of evidence associate the GO system with distinct cellular processes of *B. subtilis*, including stress-associated mutagenesis (SAM), transcriptional mutagenesis, and sporulation [31,32,33,34]. The inactivation of this system increases by 5–6 log orders, with regard to the production of His^+^ and Met^+^ (but not Leu^+^) prototrophs in nutritionally stressed cells of *B. subtilis* YB955 (*hisC952*, *metB5*, *leuC427*) [32]. Furthermore, experiments showed that Leu^+^ mutants in the strain YB955 are derived from the error-prone processing of 8-OxoG:A, A:G, and A:C mismatches caused by MutY in starved YB955 *B. subtilis* cells [31]. A recent report revealed that 8-OxoG lesions induce the generation of His^+^, Met^+^, and Leu^+^ revertants in strain YB955, and that such mutational events are mediated by the transcriptional factors Mfd and GreA [33]. In addition to its mutagenic effects, 8-OxoG was shown to affect developmental pathways in *B. subtilis*. Accordingly, a recent report showed that the processing of 8-OxoG through Mfd activated a RecA-dependent checkpoint that regulates the sporulation onset in this microorganism [34]. 

In this work, we report that growing *B. subtilis* cells deficient for a GO system increase by 3–4 orders of magnitude in terms of spontaneous Rif^R^ mutation frequency; intriguingly, this mutant strain exhibited an unusual hyperresistance to the noxious effects of the oxidizing agent hydrogen peroxide (i.e., hyperresistance to H_2_O_2_ phenotype, HPHR). 

Overall, our results unveil a novel role for 8-OxoG, beyond oxidative-induced mutagenesis; it contributes to cell fitness by reprograming the synthesis of proteins from regulons that respond to a wide diversity of cell stressors, and it confers adaptive advantages to *B. subtilis*, including hyperresistance to peroxides and transcription-acting antibiotics. 

## 2. Materials and Methods

### 2.1. Bacterial Strains, Culture Conditions, and Reagents

The strain *B. subtilis* PS832 (Wild-type *trp*^+^ revertant of strain 168 [35]), and its derivative, PERM603 (*ytkD*::*neo mutM*::*tet mutY*::*sp*. Neo^R^ Tc^R^ Sp^R^ ∆GO; [34]), were employed in this study. The routinely used growth medium was the Luria–Bertani (LB) medium. When required, neomycin (Neo) (10 μg·mL^−1^), tetracycline (Tet) (10 μg·mL^−1^), spectinomycin (Sp) (100 μg·mL^−1^), chloramphenicol (Cm) (5 μg·mL^−1^), kanamycin (Kan) (25 μg·mL^−1^), erythromycin (Ery) (5 μg·mL^−1^), or rifampin (Rif) (10 μg·mL^−1^) was added to the medium. Liquid cultures were incubated with vigorous aeration (shaking at 250 rpm) at 37 °C. Cultures on solid media were grown at 37 °C. The optical density, at 600 nm (OD_600_), of liquid cultures was monitored with a Pharmacia Ultrospec 2000 spectrophotometer (Pharmacia, Peapack, NJ, USA).

### 2.2. Cell Harvesting and Protein Extraction

*B. subtilis* strains, WT and ∆GO, were propagated in a Luria–Bertani (LB) medium to an OD_600_ of 1.0. At this point, the strain cultures were split into two subcultures; one of the subcultures was left untreated, whereas the other was supplemented with a lethal dose (fifty (LD_50_)) of hydrogen peroxide (H_2_O_2_) that was experimentally determined for each genetic background. The subcultures were incubated for an additional time of 30 min. The cultures were centrifuged at 10,000× *g* for 10 min, washed two times, and then resuspended in 10 mL of 1 X Spizizen Minimal Salts (SMS) [36]. Cell lysis was carried out by initially boiling the sample, followed by repeated freeze/thaw cycles, combined with mechanical disruption using glass beads in aqueous medium; cell debris and beads were removed by centrifugation (16,000× *g* for 10 min at 4 °C). The protein content in the cell-free extract was determined using the bicinchoninic acid method, using the Micro BCA Protein Assay Kit (Thermo Scientific; Waltham, MA, USA). Eight μg samples of each cell extract were analyzed in duplicate, using 10% sodium dodecyl (SDS), polyacrylamide gels (PAGE) that were stained with Coomassie R-250 blue dye [37]. We pooled a total of 50 μg of protein from three biological triplicates for each condition and subjected them to enzymatic digestion, following previously described protocols [38]. For LC-MS/MS, we analyzed two independent biological replicates.

### 2.3. Protein Identification and Quantitation with Mass Spectrometry Analysis LC-ESI-HDMS^E^

An aliquot from a stock solution of alcohol dehydrogenase (ADH1, Waters, Milford, MA, USA) of *Saccharomyces cerevisiae* (UniProt accession: P00330), predigested with trypsin, was added as an internal standard to all peptide samples to obtain a final concentration of 25 fmol·μL^−1^, as previously reported [39]. The resulting peptides were injected into the mass spectrometer Synapt G2-Si (Waters, Milford, MA, USA), in MS^E^ mode, to calculate the area under the curve (AUC) of the total ion chromatogram (TIC). The samples were normalized prior to injecting them into the nanoUPLC. The same number of tryptic peptides in each condition were loaded into a Symmetry C18 Trap V/M precolumn (180 μm × 20 mm, 100 Å pore size, 5 μm particle size), and they were desalted via mobile phase A (0.1% formic acid (FA) in H_2_O), and mobile phase B (0.1% FA in acetonitrile (ACN)) under the following isocratic gradient: 99.9% mobile phase A and 0.1% of mobile phase B at a flow of 5 μL·min^−1^ over 3 min. Afterwards, peptides were loaded and separated on an HSS T3 C18 column (75 μm × 150 mm, 100 Å pore size, 1.8 μm particle size). This process used an UPLC ACQUITY M-Class with the same mobile phases under the following gradient: 0 min 7% B, 121.49 min 40% B, 123.15 to 126.46 min 85% B, 129 to 130 min 7% B, at a flow of 400 nL·min^−1^ and 45 °C. The spectra data were acquired using a mass spectrometer with nanoelectrospray ionization (nanoESI) and ion mobility separation (IM-MS), via a data-independent acquisition (DIA) approach through HDMS^E^ mode (Waters, Milford, MA, USA). At the tune page, for the ionization source, parameters were set with the following values: 2.75 kV in the sampler capillar, 30 V in the sampling cone, 30 V in the source offset, 70 °C for the source temperature, 0.5 bar for the nano flow gas, and 150 L h^−1^ for the purge gas flow. Two chromatograms (low and high energy chromatograms) were acquired in positive mode, in a range of *m/z* 50–2000, with a scan time of 500 ms. The low collision energy applied was 4 eV to obtain the low-energy chromatogram, and for the high-energy chromatograms, we used a 19–55 ev collision ramp to fragment the precursor during the transfer phase.

Generated *.raw files containing MS and MS/MS spectra were deconvoluted using Progenesis QI for Proteomics v4.2 software (Waters), and they were compared with *B. subtilis* (strain 168) (downloaded from Uniprot (https://uniprot.org/, accessed on 1 May 2022), proteome ID UP000001570, 4269 proteins, 1 May 2022). Parameters used for database searching were as follows: trypsin as the cut enzyme with one missed cleavage allowed and carbamidomethyl (C) as a fixed modification, where oxidation (M), amidation (C-terminal), deamidation (Q, N), or phosphorylation (S, T, Y) were variable modifications. For the default peptide and fragment tolerance (maximum normal distribution of 10 and 20 ppm, respectively), we used 2 minimum fragment ion matches per peptide, 5 minimum fragment ion matches per protein, 1 minimum peptide match per protein, and a false discovery rate of ≤4%. Synapt G2-S*i* was calibrated with [Glu1]-fibrinopeptide, [M + 2H]^2+^ = 785.84261 at less than 1.5 ppm. The three most reliable peptides per protein (Top3) were used for absolute quantitation, according to a previously described method [40,41]. We used a Log2 Fold Change value of ±0.585, equivalent to an absolute fold change of least ±1.5 [42]. The ratio was calculated based on the absolute quantification (ng on column) of each characterized protein. The mass spectrometry proteomics data are provided as a Supplementary Materials in this manuscript.

### 2.4. Treatment of Vegetative Cells with Oxidizing Agents

Strains were propagated in a liquid LB medium at 37 °C to reach an OD_600_ of 1.0. Then, cultures were exposed to increasing doses of H_2_O_2_ or tert-Butyl hydroperoxide (*t*-BHP) for an additional period of 0.5 h. We measured cell survival by plating serial dilutions in SS 1X, comprising cells that were either unexposed or exposed to the oxidizing agents on the LB medium agar. Colonies were counted after overnight incubation at 37 °C, and CFU were determined in appropriately diluted plated samples (30 to 300 CFU). Data were reported as LD_50_ or LD_90_ values; namely, the concentrations of the oxidizing agent that killed 50% or 90% of the bacterial population. The LD values were calculated from dose–response curves (Figures S1–S3), and the experiments were repeated at least three times for each strain.

### 2.5. Determination of Mutation Frequencies

Spontaneous or H_2_O_2_-induced mutation frequencies were determined, as previously described [34]. Cells were propagated in an LB medium at 37 °C to an OD_600_ of 1.0, and they were split into two subcultures. One of the subcultures was left untreated to determine spontaneous mutagenesis, and the other was supplemented with a lethal dose 25 (LD_25_) of H_2_O_2_. The cultures were incubated at 37 °C for an additional period of 12 h. Subsequently, the cells were harvested via centrifugation (4800× *g*/10 min) at room temperature, they were washed 2 times, and resuspended in 1X SS. Cell samples (0.1 mL) were plated on six plates of LB medium and supplemented with Rif (10 μg/mL). The Rif^R^ colonies that grew after 24 h were counted. The number of cells in the bacterial culture was determined by serial dilution and the viable count on LB medium plates. The mutation frequency was reported as the average number of Rif^R^ colonies per 10^9^ viable cells. These experiments were repeated at least three times for each strain.

### 2.6. Bioinformatic Analysis

Label-free intensity analysis was selected for each individual sample. A two-sample Student’s *t* test, based on the triplicate of each sample group, was performed on log_2_-transformed intensity values. To classify proteins as variant and non-variant in a scatter plot, the Student’s *t* test difference >1 (twofold change) and *p* value < 0.05 were chosen as criteria. A heat map of the protein profiles detected in the two strains, in the absence and presence of H_2_O_2_, was performed; furthermore, an analysis was made in the “PANTHER” database (available online: http://pantherdb.org/, accessed on 1 May 2022) to classify the function of the proteins detected.

Interactions between proteins of interest were analyzed using the STRING database (https://string-db.org/, accessed on 1 May 2022). 

### 2.7. Statistical Analyses

Statistical analyses to compare the results of susceptibility with oxidizing agents and mutation frequencies between the mutant and wild-type strains were performed using one-way analysis of variance (ANOVA), followed by Tukey’s multiple comparison test, *p* < 0.05. 

## 3. Results

### 3.1. The Absence of the GO System Promotes Hypermutagenesis (HPM) and Hydrogen Peroxide Hyperresistance (HPHR) in Vegetative B. subtilis Cells

**Figure 1:**
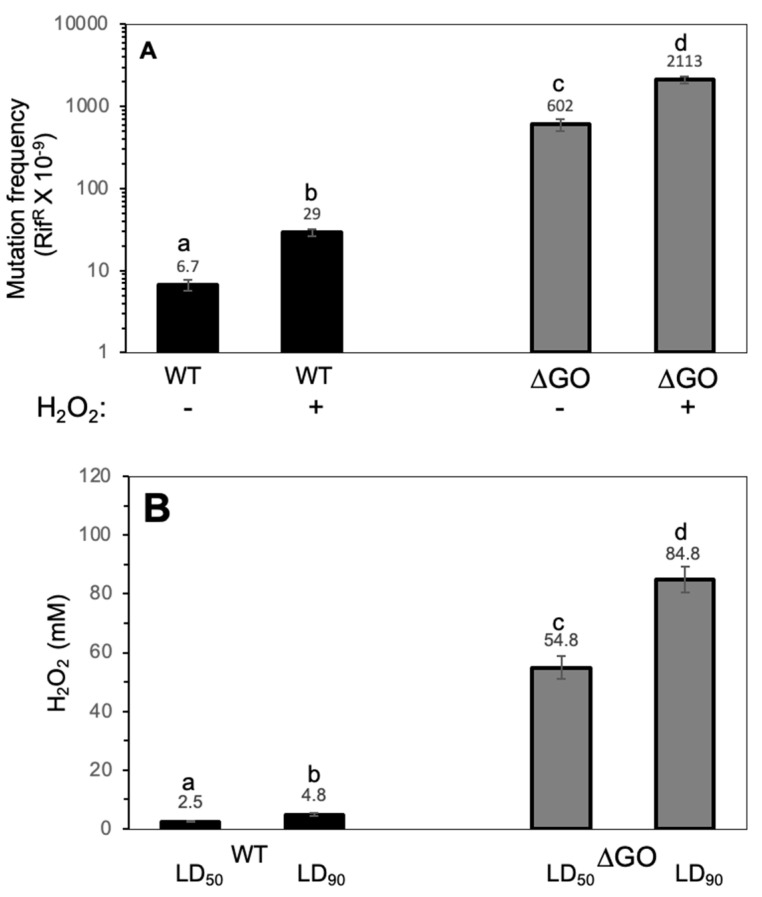
(**A**). Spontaneous- and hydrogen peroxide-promoted Rif^R^ mutation frequencies in strains of *B. subtilis* WT and ∆GO. Strains cultured to an OD_600_ of 1.0 were split into two subcultures; one of these subcultures was amended with a LD_25_ of H_2_O_2_, and the other was left as an untreated control. After a 24 h incubation period, the cultures were processed to determine mutation frequencies with regard to Rif^R^, as described in Materials and Methods. (**B**). The hydrogen peroxide susceptibility of the *B. subtilis* WT and ∆GO strains. Strains propagated with an OD_600_ of 1.0 were treated with increasing doses of hydrogen peroxide for 30 min, and LD_50s_ and LD_90s_ values were determined using dose/response curves, as described in Materials and Methods. Values represent the average of three independent experiments per triplicate ± standard deviation. Letters a–d indicate statistically significant differences between strains, as determined by one-way analysis of variance (ANOVA), followed by a Tukey’s post-hoc test; *p* < 0.05.

A previous study revealed that the simultaneous disruption of *mutM*, *mutY*, and *ytkD* (*mutTA*) increases the reversion frequencies of defective amino acid biosynthesis genes by 3–4 orders of magnitude in the stationary phase of starved *B. subtilis* cells [31,32]. In this work, we determined the impact of 8-OxoG accumulation in the growth-associated mutagenesis of *B. subtilis*. Our results demonstrated that in reference to the WT parental strain, the genetic inactivation of the GO system increases the spontaneous and H_2_O_2_-promoted mutation frequencies to Rif^R^ in growing *B. subtilis* by ~90 and ~70 fold, respectively (Figure 1A). We next investigated the ability of *B. subtilis* cells to survive the noxious effects of H_2_O_2_. Surprisingly, the lethal concentrations of H_2_O_2_ that killed 50 and 90% of the population were much higher in the GO mutant than in the parental strain. The LD_90_ value of H_2_O_2_ in the GO mutant increased by ~18 fold compared with the parental strain (i.e., 84.8 ± 8 vs. 2.5 ± 0.2) (Figure 1B). Altogether, these results indicate that the increased ability to withstand exposure to H_2_O_2_ is associated with increased mutagenesis in the GO mutant strain of *B. subtilis*.

### 3.2. The HPHR of the ΔGO Strain Is katA Dependent, but perR Independent, and Influenced by Mfd

**Figure 2:**
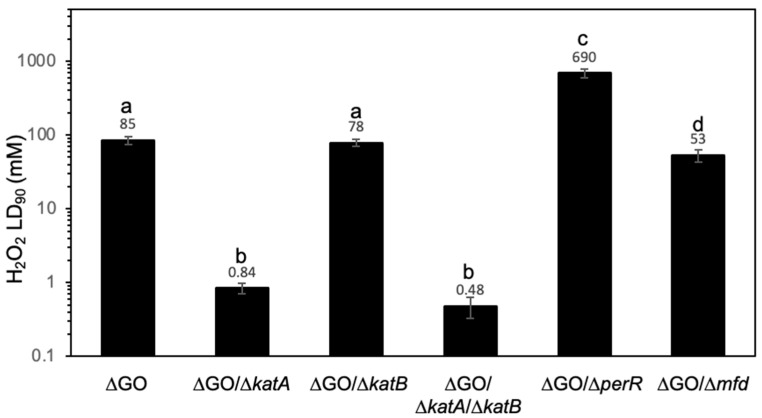
*B. subtilis* strain susceptibility to hydrogen peroxide, with distinct genotypes. The indicated strains, propagated to an OD_600_ of 1.0, were treated with increasing doses of hydrogen peroxide for 30 min; LD_90_ values were determined using dose/response curves, as described in Materials and Methods. Values represent the average of three independent experiments, per triplicate ± standard deviation. Letters a–d, indicate statistically significant differences between strains, as determined by one-way analysis of variance (ANOVA), followed by a Tukey’s post-hoc test; *p* < 0.05.

Hydrogen peroxide is detoxified in exponentially growing *B. subtilis* cells, primarily by KatA, which is encoded by the *katA* gene; the expression of this gene is controlled by the PerR repressor [12,15]. However, this microorganism also possesses KatB, whose encoding gene is regulated by the general stress sigma factor, σ^B^ [14,43]. Therefore, we first investigated the contribution of *katA* and *KatB* to the H_2_O_2_-HPHR of the ∆GO strain. To this end, *katA*, *katB*, or both were disrupted in the genetic background of ΔGO, and the resulting strains, ∆GO/∆*katA*, ∆GO/∆*katB*, and ∆GO/∆*katA*/∆*katB* were tested for their resistance to H_2_O_2_. The results showed that ∆GO KatA^−^ and ∆GO KatA^−^ KatB^−^, but not ∆GO KatB^−^ cells, exhibited a reduced tolerance to H_2_O_2_ compared with ∆GO cells (Figure 2). 

As noted above, *katA* belongs to the PerR regulon and it responds to oxidative stress. However, PerR controls the expression of other genes involved in peroxide resistance, including *ahpC-ahpF*, *mrgA*, *spx*, and *perR* [6,15]; this prompted us to investigate the contribution of all the genes in the PerR regulon to the H_2_O_2_ HPHR in the GO-deficient strain. Then, we challenged a deficient strain in both the GO system and PerR with increasing concentrations of hydrogen peroxide. As expected, the inactivation of *perR* led to the ∆GO strain exhibiting significantly increased resistance to H_2_O_2_, the LD_90_ value of this strain was 690 ± 18 compared with 85 ± 8 in the PerR replete ∆GO strain (Figure 2). Furthermore, the increased resistance to H_2_O_2_ is significantly higher in the ∆GO/Δ*perR* compared with the ∆GO/Δ*katA* strain. 

These results strongly suggest that in the GO-deficient strain (*i*) PerR is not fully activated, (*ii*) the KatA effects are PerR-independent, and (*iii*) additional factors may be involved in granting it high resistance to H_2_O_2_. 

The Mfd protein was recently shown to modulate *B. subtilis* SAM and prevent genetic damage promoted by lesions of an oxidative nature [33,44,45,46]. Here, we found that this factor influences the H_2_O_2_-HPHR of the ∆GO strain, the ∆GO ∆*mfd* cells exhibited a significant reduction in the LD_90_ to H_2_O_2_, with respect to that determined in the GO-deficient strain (53 ± 2.7 vs. 85 ± 0.7, respectively) (Figure 2).

### 3.3. Global Protein Analysis Reveals Novel Factors Contributing to the HPM and HPHR of the Strain B. subtilis ∆GO

To identify factors other than KatA, PerR, and Mfd which are involved in the HPM and HPHR of the strain *B. subtilis* ∆GO, we analyzed the global protein profile of this strain under conditions of normal growth or following exposure to H_2_O_2_. Thus, we grew cultures to the exponential phase of this mutant and its WT parental strain and treat them or not with LD_50s_ of H_2_O_2_, and we incubated them for an additional period of 30 min. The protein profiles from H_2_O_2_-treated and untreated cultures, extracted via the mechanical disruption of cells were resolved with SDS-PAGE (Supplementary Figure S4). 

The extracted proteins from each culture were analyzed via LC-MS/MS, as described in Materials and Methods. We used *B. subtilis* global databases [47,48] and identified ~500 proteins. The PANTHER database revealed that proteins involved in metabolite interconversion were numerically dominant (58%), followed by proteins of the translation machinery (17%), transporter proteins (5%), and proteins with unrelated functions (Supplementary Figure S5). Furthermore, a heat map based on protein-fold changes revealed dissimilar global protein profiles between the WT and GO-deficient strain, and between H_2_O_2_-treated and control cells in both strains (Supplementary Figure S6). 

**Figure 3:**
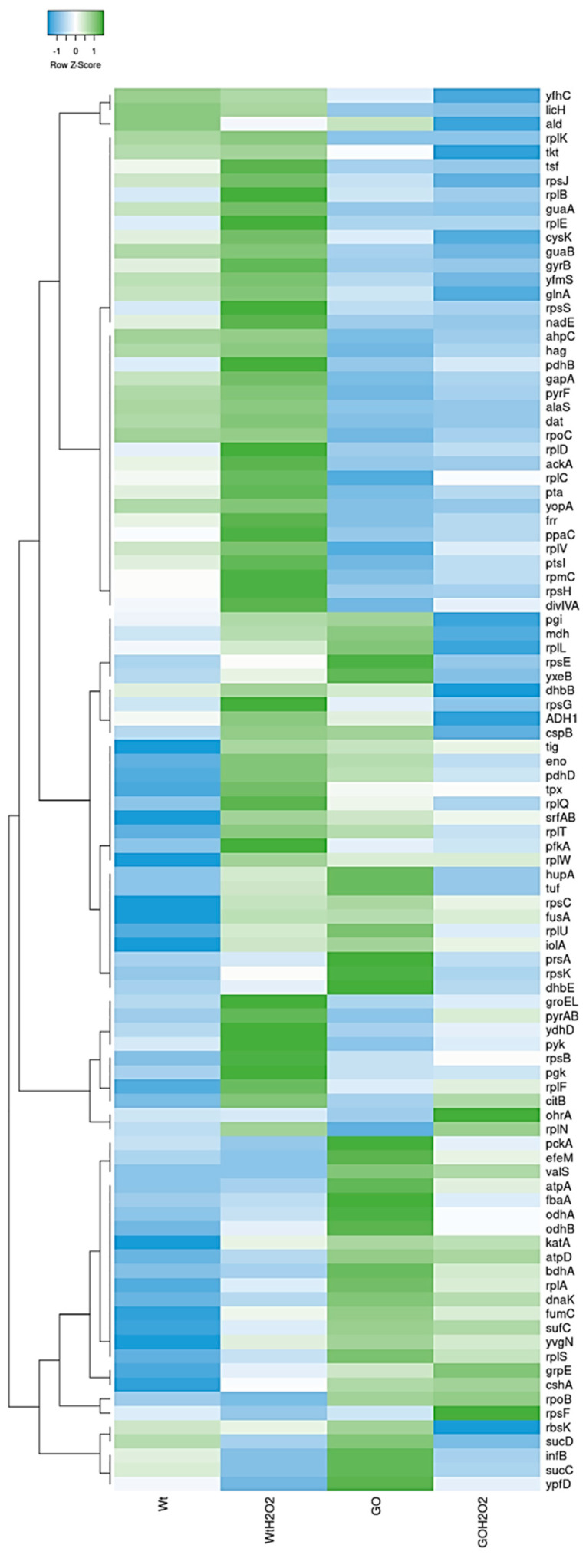
Heatmap analysis using the Log2 values of the top 100 upregulated proteins from WT and ∆GO strains that were either exposed to H_2_O_2_ or not. The graph represents the complete linkage and Spearman Rank Correlation. The Z-Score indicates differences between rows, not activation or repression.

We focused our analysis on the 100 proteins with the strongest dysregulation between the WT and ∆GO strains that were either treated with H_2_O_2_ or not. A heat map based on the Log_2_ values of these proteins confirmed the existence of dissimilar protein profiles between the WT and ∆GO strains (Figure 3). 

**Figure 4:**
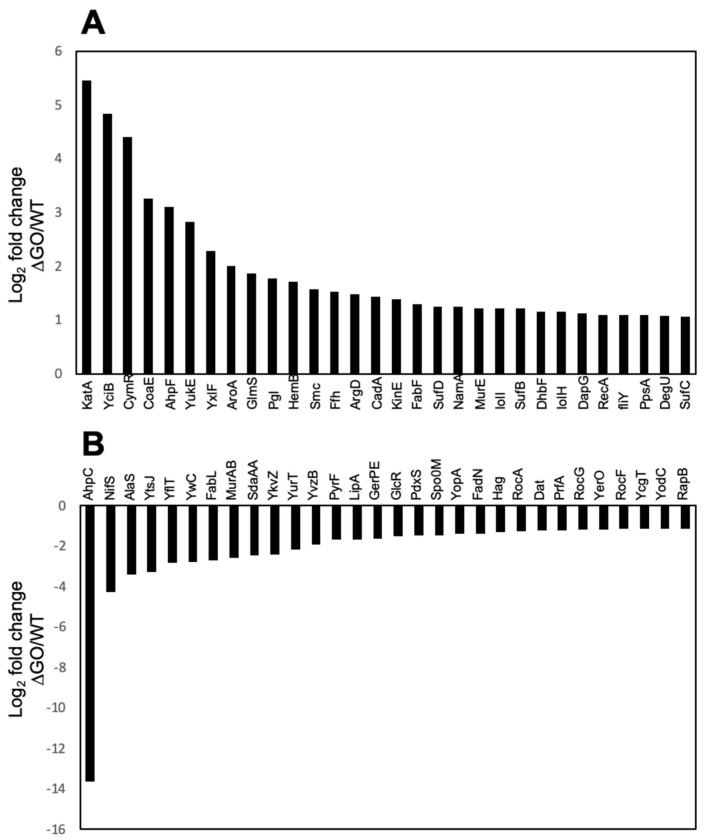
Proteins with the highest level of dysregulation in the strain, *B. subtilis* ∆GO. Bars represent the 30 proteins with the highest (**A**) and lowest (**B**) Log2 fold values detected by proteomics in the strain, *B. subtilis* ∆GO.

The fold change of the 30 proteins that exhibited the highest or the lowest synthesis in the ∆GO strain, with respect to the WT strain, were plotted and presented in Figure 4. These results revealed that proteins involved in oxidative stress, coenzyme A synthesis, cysteine metabolism, and peptidoglycan synthesis, respectively, were upregulated in the ∆GO strain (Figure 3 and Figure 4A). On the other hand, proteins involved in amino acid and lipid metabolism, as well as in general and peroxide stress, were down-regulated in the GO-deficient strain (Figure 4B). 

**Figure 5:**
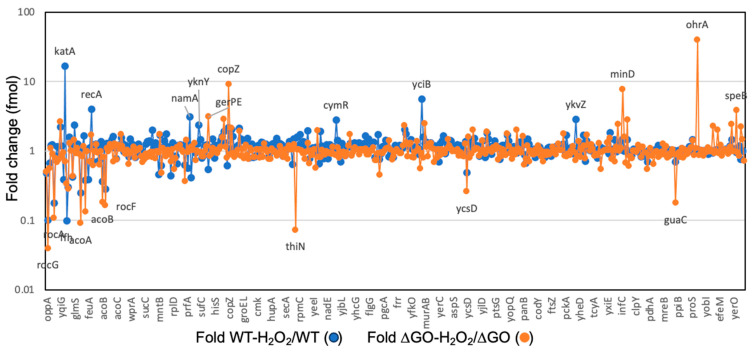
Hydrogen peroxide elicits differential protein profiles in the WT and ∆GO *B. subtilis* strains. Protein abundance values (fmol) were plotted as fold changes for the WT and ∆GO strains exposed to H_2_O_2_ versus the same untreated strains. Proteins with the highest and lowest fold change values are indicated in the figure.

Treatment with H_2_O_2_ elicited different protein profiles in the WT and ∆GO strains (Figure 3 and Supplementary Figure S6). Thus, proteins like KatA, AhpC/AhpF, and RecA, from the PerR and SOS response [15,49,50,51], were induced by H_2_O_2_ in the WT strain (Figure 5). On the other hand, OhrA and CopZ, whose encoding genes were under the transcriptional control of OhrR and CopR, respectively [16,52], were upregulated by H_2_O_2_ in the GO-deficient strain (Figure 5). 

To further understand the atypical phenotypes of the ∆GO strain and its response to H_2_O_2_, we analyzed highly dysregulated proteins associated with different types of cellular stresses (Table 1). We found KatA to be the protein with the highest positive fold change in the GO-deficient strain (Figure 4A and Table 1). These results agreed with our assays measuring expression in a strain containing a *lacZ* fusion inserted in the *katA* locus; β-galactosidase activity was 18 times higher in the ∆GO strain than in the WT strain (namely, 312 ± 12.3 vs. 68 ± 7.9). Of note, although the levels of AhpF (one of the subunits of the PerR-regulated alkyl hydroperoxide reductase (AHPR) [15]) exhibited a high fold change value (i.e., 3.1), AhpC (the other subunit of this enzyme and the peroxide stress protein OhrA [16]) showed an opposite effect exhibiting lower Log2 folds; namely, −13.7 and −0.76, respectively (Table 1). These results suggest that the increased resistance to H_2_O_2_ in the GO-deficient strain does not require AhpC/AhpF-dependent AHPR to counteract the noxious effects of organic peroxides [53,54]. 

Further factors involved in redox and disulfide stresses, including CymR, SufA, SufB, and SufC, respectively, are upregulated at different levels in the ∆GO strain (Table 1). CymR is a repressor involved in the metabolism of cysteine [55], whereas SufA-C are scaffold proteins that play essential roles during the synthesis of iron–sulfur clusters [56]. 

Proteins like the putative peptidyl transferases, YciB, which are involved in peptidoglycan biosynthesis (PG) [57,58], and the DNA binding protein, HupA (Hbs), which regulates the compaction of the nucleoid [59,60], were also found to be upregulated in the ∆GO strain (Table 1). The TrxA, YceD, and SmC proteins, which are involved in distinct cell stress responses [61], were also dysregulated in the GO-deficient strain (Table 1). The expression of the gene-coding for these proteins is under the control of the SigB and Fur factors [62,63]. 

In the ∆GO strain, we compared the proteins with a dysregulated status in H_2_O_2_-treated and untreated cultures. Interestingly, H_2_O_2_ treatment causes KatA levels to decrease, but OhrA concentration increases compared with the untreated cells (Table 1). A similar response was observed for YciB and HupA in the ∆GO strain (Table 1); H_2_O_2_-treated cells displayed lower levels of these proteins than untreated cells. These results strongly suggest that in addition to KatA, proteins whose syntheses are controlled by Zur, Fur, RecA, and SigB factors, as well as the stringent response, are involved in the HPM and HPHR phenotypes of the ∆GO strain.

### 3.4. YciB, OhrA, and SigB Contribute to the HPM and HPHR of the Strain, B. subtilis ∆GO

**Figure 6:**
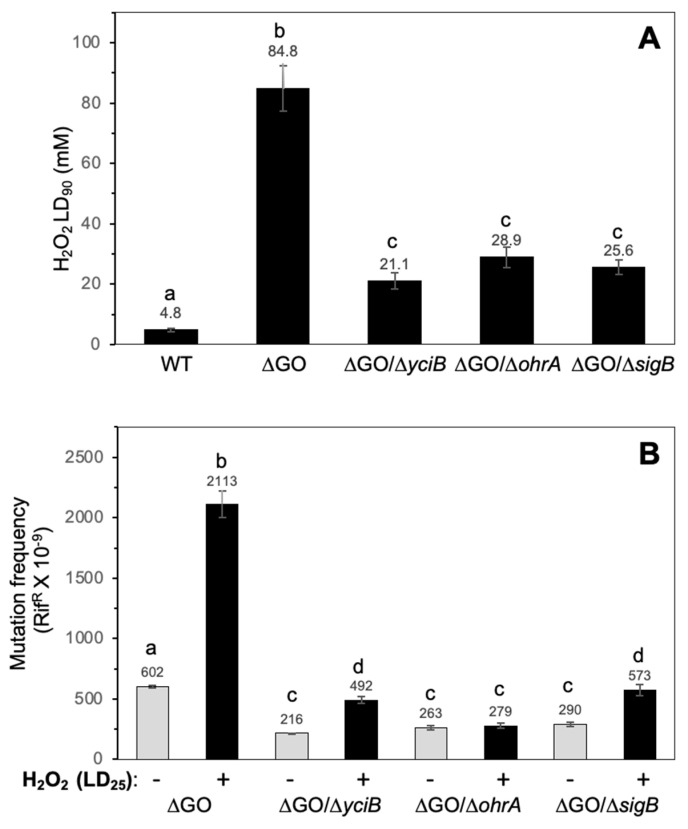
(**A**). *B. subtilis* strain susceptibility to hydrogen peroxide, with distinct genotypes. The indicated strains with an OD_600_ of 1.0 were treated with increasing doses of hydrogen peroxide for 30 min; LD_90s_ values were determined using dose/response curves, as described in Materials and Methods. Values represent the average of three independent experiments, per triplicate ± standard deviation. Letters a–c indicate statistically significant differences between strains, as determined by a one-way analysis of variance (ANOVA) followed by a Tukey’s post-hoc test; *p* < 0.05. (**B**). Spontaneous- and hydrogen peroxide-promoted Rif^R^ mutation frequencies of *B. subtilis* strains with different genotypes. Strains cultured to an OD_600_ of 1.0 were split into two subcultures, one of these subcultures was amended with a LD_25_ of H_2_O_2_, and the other was left as an untreated control. After a 24 h incubation period, the cultures were processed to determine mutation frequencies in Rif^R^, as described in Materials and Methods. Values represent the average of three independent experiments, per triplicate ± standard deviation. Letters a–d indicate statistically significant differences between strains, as determined by a one-way analysis of variance (ANOVA) followed by a Tukey’s post-hoc test; *p* < 0.05.

The contribution of the peptidyl transferase, YciB, the organic peroxide reductase, OhrA, and the general stress sigma factor, SigB, to stress phenotypes of the GO-deficient strain, was measured in strains with disruptions to *yciB*, *ohrA*, and *sigB*. Results from inactivation curves, and the determination of lethal doses revealed that the disruption of *yciB, ohrA*, and *sigB* significantly reduces the H_2_O_2_-hyperresistence of *B. subtilis* ΔGO cells (Figure 6A). The ∆GO strain exhibited a LD_90_ of 84.8 ± 4.5, compared with 21.16 ± 2.3, 28.97 ± 4.3, and 25.6 ± 2.1 in the ∆GO/∆*yciB*, ∆GO/∆*ohrA*, and ∆GO/∆*sigB* strains, respectively (Figure 6A).

We also determined the spontaneous and hydrogen peroxide-promoted mutagenesis in strains that lacked the GO system, and disruptions in the genes *yciB*, *ohrA*, or *sigB*. In reference to the GO-deficient strain, the spontaneous Rif^R^ mutagenesis decreased ~3 times in the ∆GO/∆*yciB*, ∆GO/∆*ohrA*, and ∆GO/∆*sigB* mutants (Figure 6B). Furthermore, in reference to the ∆GO strain, the H_2_O_2_-induced mutagenesis decreased by 4.2, 7.5, and 3.7 times in the same background, with disruptions occurring in *yciB*, *ohrA*, or *sigB*, respectively (Figure 6B). Altogether, these results indicate that SigB, YciB, and OhrA contribute to the HPHR and HPM phenotypes of the ∆GO strain.

### 3.5. H_2_O_2_ Pretreatment Increases the Resistance of the GO-Deficient Strain to t-BHP

**Figure 7:**
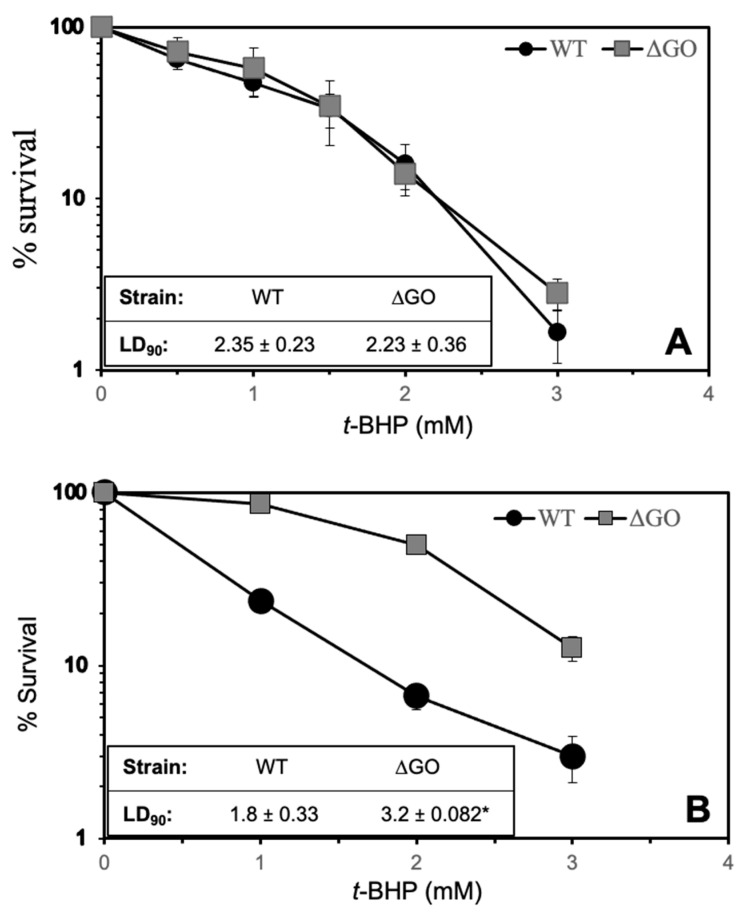
*B. subtilis* WT and ∆GO strain susceptibility to *t*-BHP (**A**). Strains with an OD_600_ of 1.0 were treated with increasing doses of *t*-BHP for 30 min; LD_90s_ values were determined from the dose/response graphs, as described in Materials and Methods. Values represent the average of three independent experiments, per triplicate ± standard deviations. (**B**). The indicated strains were treated with an OD_600_ of 0.8, which was amended with a LD_25_ of H_2_O_2_, and then allowed to increase to an OD_600_ of 1.0. At this point, the strains were treated with increasing doses of *t*-BHP for 30 min; LD_90s_ values were determined using the dose/response graphs, as described in Materials and Methods. Values represent the average of three independent experiments, per triplicate ± standard deviations. Asterisks (*) indicate statistically significant differences between LD_90_ values and strains, as determined by a one-way analysis of variance (ANOVA) followed by a Tukey’s post-hoc test; *p* < 0.05.

As noted above, the levels of AhpC and OhrA, which are key factors that confer protection from organic peroxides [16,53,54], are downregulated in *B. subtilis* cells that are deficient in the GO system; this result prompted us to test the ability of this strain to withstand treatment with other oxidants. To this end, growing cultures of the WT and the GO-deficient strains were treated with increasing doses of tert-butyl hydroperoxide (t-BHP). These assays revealed no significant differences between either strain, with regard to the harmful effects of this organic peroxide (Figure 7A). However, pretreatment of the ∆GO strain with H_2_O_2_, which upregulates OhrA (Table 1), increased its resistance to *t*-BHP compared with the WT strain (Figure 7B). It is possible that pretreatment with hydrogen peroxide inactivates the repressor effect of OhrR over *ohrA*, and increases the levels of its encoding product, OhrA.

### 3.6. The Absence of a GO System Confers B. subtilis Resistance to Antibiotics That Target Transcription but Not DNA Replication

**Figure 8:**
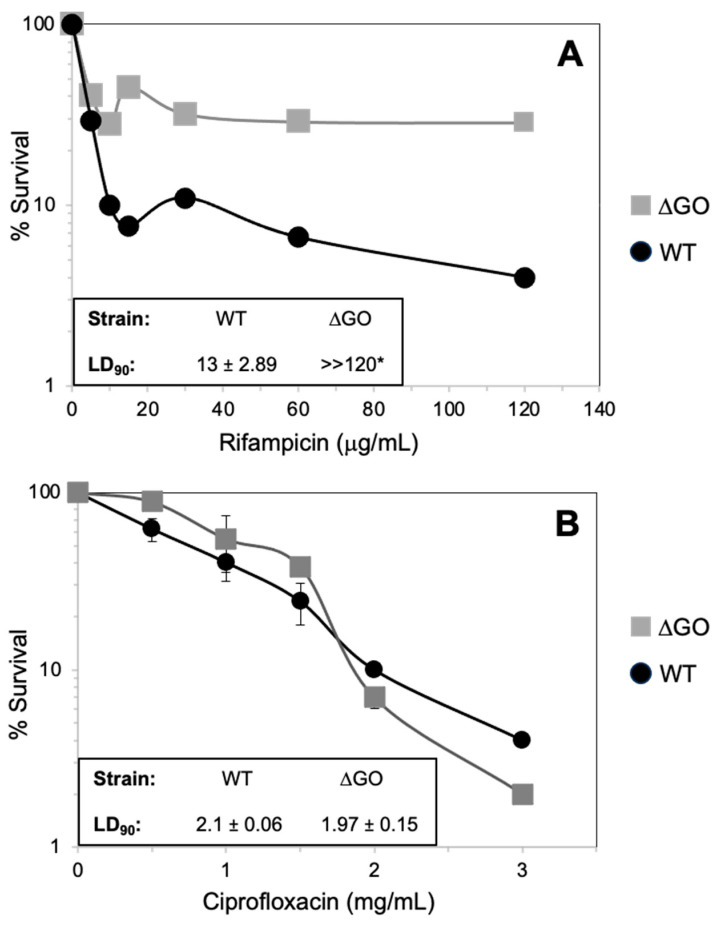
*B. subtilis* WT and ∆GO strain susceptibility to rifampicin and ciprofloxacin. Strains with an OD_600_ of 1.0 were treated with increasing doses of rifampicin (**A**) or ciprofloxacin (**B**), at the indicated concentrations, for 30 min; LD_90s_ values were determined using the dose/response graphs, as described in Materials and Methods. Values represent the average of three independent experiments, per triplicate ± standard deviations. Asterisks (*) indicate significant differences between LD_90_ values and strains, as determined by a one-way analysis of variance (ANOVA) followed by a Tukey’s post-hoc test; *p* < 0.05.

As shown in this work, the synthesis of factors that is expressed in response to different cell stress responses is dysregulated in the GO-deficient strain. We determined whether this atypical physiological status in the ∆GO strain is associated with antibiotic resistance. Exponentially growing cultures in the WT and GO-deficient strain were independently challenged with increasing concentrations of rifampicin (Rif) or ciprofloxacin (Cyp), and the fraction of surviving cells was determined by viable counts. The results revealed a marked resistance of the ∆GO strain to Rif, but not to Cyp, with respect to the WT parental strain (Figure 8). Ninety percent of the cell population of the parent strain was inactivated at a concentration of 13 ± 2.89; however, 30% the ∆GO cells were viable at a concentration of 120 μg/mL of this transcription targeting antibiotic (Figure 8A). Conversely, 90% of the cells in both strains were inhibited by similar Cyp concentrations (Figure 8B). Altogether, these results indicate that the inactivation of the GO system which leads to the accumulation of 8-OxoG [63] confers resistance to rifampicin in *B. subtilis*.

## 4. Discussion

Previous reports suggested that disabling the GO system increases genetic diversity in *B. subtilis* and the likelihood of escaping growth-limiting conditions [30,31,32]. In this work, we report that a *B. subtilis* strain that was deficient for the GO system, and prone to accumulating genomic 8-OxoG lesions [63], increased in terms of its spontaneous mutagenesis. It also exhibited an unusual hyperresistance to hydrogen peroxide and antibiotics that suppress RNA synthesis. Our proteomic and genetic analyses strongly suggest that these phenotypes are associated with the gratuitous activation of factors that counteract cellular responses controlled by the PerR, OhrA, Zur, and SigB regulators.

A previous report revealed that Tn10 insertions in *ahpC*, encoding one of the two subunits of the alkyl hydroperoxide reductase, activate the expression of the full PerR operon, including KatA. Based on zone inhibition assays, the authors reported a marked increase in H_2_O_2_ resistance in *B. subtilis* [54]. Here, we report a *B. subtilis* strain, that is defective in terms of its ability to process 8-OxoG lesions, exhibits increased KatA protein levels, via the increased transcription of *katA*, and it is highly resistant to hydrogen peroxide. 

A marked increase in KatA synthesis was also reported in a *B. subtilis* strain bearing a PerR-null allele; however, all components of the PerR regulon were upregulated in this mutant strain [64]. In contrast, in the ∆GO strain, only AhpF (but not AhpC), HemB, and KatA, were found to be upregulated. Our results also revealed that in the GO-deficient strain, PerR is completely functional as the disruption of its encoding gene induces a dramatic increase in H_2_O_2_ resistance (Figure 2). Furthermore, the expression levels of a transcriptional *katA-lacZ* fusion, inserted in the *katA* locus, increased from 312 ± 12 to 658 ± 36.8 in the ∆GO strain, following the disruption of *perR*. Taken together, these results supported the following concepts: in the GO-deficient genetic background, (*i*) the PerR regulon is not gratuitously upregulated, (*ii*) additional factors are involved in its hyperresistance to H_2_O_2_, and (*iii*) a mechanism(s) that is independent of PerR, increases the expression of *katA*. In support of these notions, in addition to KatA, factors that are induced by distinct types of cell stress, including OhrA, YciB, HupA, YfiT, YceD, DnaK, and GrpE, were found to be dysregulated in the GO-deficient strain. YciB stands out among these factors because its increased synthesis was as pronounced as in KatA (Table 1). Distinct functions have been attributed to this putative D-L transpeptidase protein, whose coding gene belongs to the *B. subtilis* Zur regulon, including zink transport, cell wall biosynthesis and cellular division [57,58]. We speculate that the levels of YciB increased in response to the accumulation of 8-OxoG and ROS-induced damage [63], which impact the integrity of the cell wall. This hypothesis is supported by reports demonstrating that D-L transpeptidases, like YciB, play important roles in maintaining the homeostasis of the bacterial cell envelope during ROS-promoted stress or exposure to β-lactam antibiotics [65]. In the bacterium *E. coli*, expression of *ldtD*, the homologue of *B. subtilis yciB*, is controlled by the two-component system, Cpx/CpA, which is activated under conditions that cause stress to its cell envelopes [66,67]. Here, we found that the levels of YciB, but no other Zur-regulated proteins, is increased in *B. subtilis* cells that lack a functional GO system. These findings suggest that *B. subtilis* cells, which accumulate 8-OxoG, activate a yet-to-be defined mechanism that positively upregulates the levels of YciB. 

In the GO-deficient strain, the increased synthesis of CymR, the transcriptional repressor for genes controlling cysteine homeostasis, was detected [55]. In *B. subtilis*, CymR negatively regulates the expression of genes required for the transport and synthesis of cystine and the assimilation of sulfonate [55]. It has been postulated that in the presence of cysteine, CymR and CysK (which is required for cysteine synthesis) [68] interact to establish a quaternary (Csk_2_/CymR_2_) complex that represses the expression of genes belonging to CymR regulon [55,68]. Here, we found that in reference to the WT strain, the concentration of CymR increased by ~20 times in the GO deficient strain (Table 1). Although no differences in CysK levels were found between either strain, the level in both strains was three times higher than those observed with CymR (Table 1). Therefore, the concentration of both proteins is sufficient to generate the active Csk_2_/CymR_2_ complex and increase resistance to H_2_O_2_ in the GO-deficient strain. In support of this notion, a recent report revealed that CymR deficiency increases *B. subtilis* sensitivity to oxidizing agents such as hydrogen peroxide, paraquat, and disulfide, and that it is promoted by tellurite [69]. In our work, it was also found that NifS (IcsC), an enzyme that transfers sulfur from cysteine to SH-carriers (like pyridoxal phosphate) in order to generate alanine, is down-regulated in the strain that lacks the GO system (Figure 4) [48]. Altogether, these observations strongly suggest that NifS down-regulation and the CymR-dependent repression of cysteine directs cysteine during CoA synthesis, presumably for the CoA-lation of OhrR. These reactions lead to the transcriptional derepression of the *ohrA* gene and increases in the levels of the organic peroxide reductase, OhrA.

As noted previously, the levels of AhpF, the large subunit of *B. subtilis* AHPR involved in detoxifying organic peroxides, was upregulated in the GO-deficient strain; remarkably, a dramatic reduction in the cellular concentration of the small subunit of this enzyme, AhpC, was detected in this mutant strain. Here, we showed that the protein levels of AhpC and AhpF increased in cultures exposed to H_2_O_2_ in the WT (Table 1), an expected result, as the genes encoding these proteins are under the control of the repressor, PerR [15,49]. Therefore, a non-described mechanism reduces the levels of AhpC, and consequently, reduces AhpC/AhpF-dependent AHPR activity in the ∆GO strain. Furthermore, the disparate levels in the AHPR subunits and decreased activity do not result in an increase in susceptibility to the organic peroxide, *t*-BHP (Figure 7A). It is possible that additional proteins possessing AHPR activity [70] can compensate for the divergent levels of AhpC and AhpF, and thus, prevent a drop in sensitivity to *t*-BHP.

Our proteomic analysis also revealed that CoaE, the enzyme that catalyzes the last step in the synthesis of Coenzyme A (CoA), was upregulated in the ∆GO strain (Figure 4 and Table 1). CoA is a key cofactor for cellular metabolic pathways, however, our work and previous evidence [71] note that this cofactor prevents oxidative damage in bacteria. Of note, the CoA factor carries S-thiolation over redox active cysteine residues of transcription factors like PerR, CtsR, and OhrR) which regulates gene expression during oxidative stress [71]. The consequences of CoA-lation were elucidated for *B. subtilis* OhrR, a protein that represses the transcription of *ohrA*, which encodes a thiol-dependent peroxidase that counteracts the toxicity of organic hydroperoxides like cumene- hydroperoxides (CMHP) and tert-butyl-hydroperoxide (*t*-BHP) [72]. Therefore, in the ∆GO strain, the higher levels of CoaE can be involved in increasing the concentration of CoA to prevent oxidative stress. Although the proteomic analysis did not detect differences in OhrA levels between the ∆GO and the parental WT strain, the presence of H_2_O_2_ in the culture medium increased the levels of OhrA in the former, but not in the latter (Table 1). In line with these observations, our results revealed that H_2_O_2_ pretreatment significantly increased the resistance of the GO-deficient strain to the noxious effects of *t*-BHP, with respect to the WT strain (Figure 7B). 

Several factors belonging to the general stress regulon exhibited a dysregulated status in the ∆GO strain, including YceE and YcdE, which are required to survive ethanol stress and low temperatures [73,74]. Remarkably, Trx, another general stress protein that fulfills an essential role in cleaving CoA from proteins modified with this cofactor, including OhrR [75], was also upregulated in this mutant strain. Therefore, the deCoA-lation of OhrA, and possibly other protein targets of this posttranslational modification, is required to maintain the ability of the ∆GO strain to contend with the noxious effects of H_2_O_2_. Our results supported this notion as the disruption of the σ^B^ encoding gene reduced the HPHR of this mutant strain (Figure 6A). 

Notably, the genetic inactivation of the GO system, which leads to the accumulation of 8-OxoG [63], conferred adaptive advantages to *B. subtilis*, including hyperresistance to the antibiotic, rifampicin. In line with this, it has been shown that 8-OxoG and other oxidized bases promote base substitutions in *rpoB*, giving rise to rifampicin resistance in *E. coli* and *B. subtilis* [76]. However, disruption to the GO system did not promote hyperresistance to fluoroquinolone in *B. subtilis* (Figure 8B), suggesting that DNA lesions other than 8-OxoG are associated with this phenotype. In support of this hypothesis, in *Bacillus anthracis*, the hyperresistance to ciprofloxacin is associated with base deamination events that generate cytosine from thymine, and facilitate guanine to adenine transitions in the *gyrA* gene [77]. Here, we found that RpoB, the core RNA polymerase subunit, was upregulated in the GO-deficient strain (Figure 3 and Table 1). Therefore, it is feasible that transcription-associated mutations occurring in *rpoB* can elicit alterations to the program of genetic expression in the ∆GO strain, thus positively impacting its ability to adapt to a diverse range of stress conditions [78,79,80]. 

Overall, the evidence presented in this work links the ROS-promoted lesion, 8-OxoG, with the differential synthesis of proteins belonging to distinct transcriptional regulons, allowing *B. subtilis* to contend with the noxious effects of ROS-generating factors. As shown in this work, these processes can be subjected to regulation with Mfd (Figure 2), a transcription factor commonly associated with the repair of bulky DNA lesions in bacteria [81]. However, recent evidence has revealed that Mfd functions as a global regulator of gene transcription and cell differentiation in *B. subtilis* [34,82]. Therefore, elucidating the Mfd-dependent mechanisms that cells employ to directly or indirectly regulate the expression of the factors associated with the HPM and HPHR phenotypes will contribute to understanding the novel aspects of bacterial physiology in stressed bacterial cells.

Although our work unveils a connection between the 8-OxoG lesion and the ability of *B. subtilis* to adapt to HPM and withstand peroxide and antibiotic stresses, the mechanism(s) involved in regulating this response remain to be discovered. Experiments aimed at investigating whether the chemical nature of 8-OxoG, the proteins involved in its recognition/repair, or the products concerning its processing, constitute the signal that activates the GO-regulon are required.

## 5. Conclusions

Experimental evidence shown in this report uncovers novel roles for the ROS-promoted 8-OxoG lesion, beyond oxidative stress-promoted mutagenesis in the Gram-positive microorganism *B. subtilis*, including (*i*) the activation of different stress-associated protein profiles to counteract the genotoxic and cytotoxic impact of organic peroxides, and (*ii*) the induction of increased resistance to transcription-acting antibiotics. 

## Figures and Tables

**Table 1:** Abundance and fold changes of stress-associated proteins from *B. subtilis* strains, ∆GO and WT.

Protein	Cell Function	Essential	Protein Abundance (fmol)				∆GO/WT		∆GO H_2_O_2_/GO	
			WT	∆GO	WT H_2_O_2_	∆GO H_2_O_2_	Max Fold	Log_2_ Max Fold	Max Fold	Log_2_ Max Fold
KatA	Oxidative stress	No	5.21	227.71	87.7	161	43.5	5.45	0.7	−0.51
YciB	Zur regulon	No	0.54	15.6	3.02	14.37	28.9	4.84	0.92	−0.12
CoaE	Peroxide stress	Yes	0.67	6.39	0.26	6.02	9.5	3.25	0.94	−0.09
AhpF	Oxidative stress	No	19.55	169	39.2	137.36	8.64	3.1	0.81	−0.3
AhpC	Oxidative stress	No	250.3	0.02	478.3	0.06	7.60 × 10^−5^	−13.7	3	1.58
OhrA	Oxidative stress	No	0.42	0.23	0.45	9.3	0.53	−0.76	40.43	5.3
CymR	Redox stress and stringent response	No	1.32	27	3.7	24	20.4	3.2	0.88	−0.02
CysS	Redox stress and stringent response	Yes	7.32	8.25	8.42	7.87	1.12	0.16	0.95	−0.07
CysK	Redox stress and stringent response	No	94	91	97.8	89.5	0.96	−0.06	0.98	−0.03
SufB	Redox stress	Yes	26.7	62.1	31.4	56.1	2.32	1.21	0.9	−0.12
SufD	Redox stress	Yes	25.5	61.2	30.9	58.86	2.4	1.26	0.96	−0.07
SufC	Redox stress	Yes	34.6	77.1	53.1	73.8	2.2	1.13	0.95	−0.07
SufS	Redox stress	Yes	10.8	16	17.6	17.2	1.5	0.58	1.07	0.1
YutI(Nfu)	Redox stress	No	15.8	14.3	16.6	13.9	0.9	−0.15	0.97	−0.08
RecA	SOS response	No	2.7	5.8	10.9	10	2.1	1.07	1.7	0.76
Hupa	SOS response		303.4	401.5	359.1	306.4	1.32	0.4	0.76	−0.39
YceD	General stress	No	21.12	34.9	18.9	32.1	1.65	0.72	0.91	−13
TrxA	General stress	Yes	1.1	2.34	1.9	1.9	1.99	0.99	0.82	−0.28
YceE	General stress	No	19.2	28.22	25.3	24.9	1.46	0.55	0.88	−0.02
YflT	General stress	No	13.3	1.9	12.4	3.82	0.14	−1.32	2	1
SmC	Stringent response		3	9.3	4.9	8.76	3.07	1.63	0.94	−0.09
RpoB	Stringent response		59.54	68.71	58.57	69.2	1.15	0.2	1	0

## Data Availability

All data are provided as Supplementary Materials.

## Supplementary Materials

The following supporting information can be downloaded at: https://www.mdpi.com/article/10.3390/antiox13030332/s1, Figures S1–S3: Hydrogen peroxide susceptibility of *B. subtilis* strains with distinct genotypes. Figure S4: SDS-PAGE analysis of protein cell extracts from strains *B. subtilis* WT and ∆GO. Figure S5: Functional distribution of differentially expressed class proteins between the WT and ∆GO *B. subtilis* strains subjected to hydrogen peroxide stress. Figure S6: Complete proteome differences between strains *B. subtilis* WT and ∆GO. Supplemental proteomic data. The mass spectrometry proteomics data are provided as supplementary material.

## References

[1] Imlay J.A. (2008). Cellular defenses against superoxide and hydrogen peroxide. Ann. Rev. Biochem..

[2] Imlay J.A. (2019). Where in the world do bacteria experience oxidative stress?. Environ. Microbiol..

[3] Bonilla C.Y. (2020). Generally Stressed Out Bacteria: Environmental Stress Response Mechanisms in Gram-Positive Bacteria. Integr. Comp. Biol..

[4] Imlay J.A. (2013). The molecular mechanisms and physiological consequences of oxidative stress: Lessons from a model bacterium. Nat. Rev. Microbiol..

[5] Fasnacht M., Polacek N. (2021). Oxidative stress in bacteria and the central dogma of molecular biology. Front. Mol. Biosci..

[6] Faulkner M.J., Helmann J.D. (2011). Peroxide stress elicits adaptive changes in bacterial metal ion homeostasis. Antioxid. Redox. Signal.

[7] Henle E.S., Linn S. (1997). Formation, prevention, and repair of DNA damage by iron/hydrogen peroxide. J. Biol. Chem..

[8] Imlay J.A. (2006). Iron-sulphur clusters and the problem with oxygen. Mol. Microbiol..

[9] Keyer K., Imlay J.A. (1996). Superoxide accelerates DNA damage by elevating free-iron levels. Proc. Natl. Acad. Sci. USA.

[10] Henle E.S., Han Z., Tang N., Rai P., Luo Y., Linn S. (1999). Sequence-specific DNA cleavage by Fe^2+^-mediated fenton reactions has possible biological implications. J. Biol. Chem..

[11] Sies H., Jones D.P. (2020). Reactive oxygen species (ROS) as pleiotropic physiological signalling agents. Nat. Rev. Mol. Cell Biol..

[12] Helmann J.D., Wu M.F.W., Gaballa A., Kobel P.A., Morshedi M.M., Fawcett P., Paddon C. (2003). The global transcriptional response of *Bacillus subtilis* to peroxide stress is coordinated by three transcription factors. J. Bacteriol..

[13] Price C.W. (2014). General Stress Response. Bacillus Subtilis and Its Closest Relatives: From Genes to Cells.

[14] Nannapaneni P., Hertwig F., Depke M., Hecker M., Mäder U., Völker U., Steil L., van Hijum S.A.F.T. (2012). Defining the structure of the general stress regulon of *Bacillus subtilis* using targeted microarray analysis and random forest classification. Microbiology.

[15] Pinochet-Barros A., Helmann J.D. (2018). Redox sensing by Fe^2+^ in bacterial Fur family metalloregulators. Antioxid. Redox Signal..

[16] Fuangthong M., Atichartpongkul S., Mongkolsuk S., Helmann J.D. (2001). OhrR is a repressor of *ohrA*, a key organic hydroperoxide resistance determinant in *Bacillus subtilis*. J. Bacteriol..

[17] Leichert L.I.O., Scharf C., Hecker M. (2003). Global characterization of disulfide stress in *Bacillus subtilis*. J. Bacteriol..

[18] Steenken S., Jovanovic S.V. (1997). How easily oxidizable is DNA? One-electron reduction potentials of adenosine and guanosine radicals in aqueous solution. J. Am. Chem. Soc..

[19] Kino K., Hirao-Suzuki M., Morikawa M., Sakaga A., Miyazawa H. (2017). Generation, repair and replication of guanine oxidation products. Genes Environ..

[20] Saito I., Nakamura T., Nakatani K., Yoshioka Y., Yamaguchi K., Sugiyama H. (1998). Mapping of the hot spots for DNA Damage by one-electron oxidation: Efficacy of GG doublets and GGG triplets as a trap in long-range hole migration. J. Am. Chem. Soc..

[21] Wood M.L., Dizdaroglu M., Gajewski E., Essigmann J.M. (1990). Mechanistic studies of ionizing radiation and oxidative mutagenesis: Genetic effects of a single 8-hydroxyguanine (7-hydro-8-oxoguanine) residue inserted at a unique site in a viral genome. Biochemistry.

[22] Shibutani S., Takeshita M., Grollman A.P. (1991). Insertion of specific bases during DNA synthesis past the oxidation-damaged base 8-oxodG. Nature.

[23] De Rosa M., Johnson S.A., Opresko P.L. (2021). Roles for the 8-Oxoguanine DNA repair system in protecting telomeres from oxidative stress. Front. Cell Dev. Biol..

[24] Michaels M.L., Miller J.H. (1992). The GO system protects organisms from the mutagenic effect of the spontaneous lesion 8-hydroxyguanine (7,8-dihydro-8-oxoguanine). J. Bacteriol..

[25] Michaels M.L., Cruz C., Grollman A.P., Miller J.H. (1992). Evidence that MutY and MutM combine to prevent mutations by an oxidatively damaged form of guanine in DNA. Proc. Natl. Acad. Sci. USA.

[26] Maki H., Sekiguchi M. (1992). MutT protein specifically hydrolyses a potent mutagenic substrate for DNA synthesis. Nature.

[27] Ramírez M.I., Castellanos-Juárez F.X., Yasbin R.E., Pedraza-Reyes M. (2004). The *ytkD*(*mutTA*) gene of *Bacillus subtilis* encodes a functional antimutator 8-Oxo-(dGTP/GTP)ase and is under dual control of Sigma A and Sigma F RNA polymerases. J. Bacteriol..

[28] Castellanos-Juárez F.X., Álvarez-Álvarez C., Yasbin R.E., Setlow B., Setlow P., Pedraza-Reyes M. (2006). YtkD and MutT protect vegetative cells but not spores of *Bacillus subtilis* from oxidative stress. J. Bacteriol..

[29] Tajiri T., Maki H., Sekiguchi M. (1995). Functional cooperation of MutT, MutM and MutY proteins in preventing mutations caused by spontaneous oxidation of guanine nucleotide in *Escherichia coli*. Mutat. Res..

[30] Gómez-Marroquín M., Vidales L.E., Debora B.N., Santos-Escobar F., Obregón-Herrera A., Robleto E.A., Pedraza-Reyes M. (2015). Role of *Bacillus subtilis* DNA glycosylase MutM in counteracting oxidatively induced DNA damage and in stationary-phase-associated mutagenesis. J. Bacteriol..

[31] Debora B.N., Vidales L.E., Ramírez R., Ramírez M., Robleto E.A., Yasbin R.E., Pedraza-Reyes M. (2011). Mismatch repair modulation of MutY activity drives *Bacillus subtilis* stationary-phase mutagenesis. J. Bacteriol..

[32] Vidales L.E., Cárdenas L.C., Robleto E., Yasbin R.E., Pedraza-Reyes M. (2009). Defects in the error prevention oxidized guanine system potentiate stationary-phase mutagenesis in *Bacillus subtilis*. J. Bacteriol..

[33] Leyva-Sánchez H.C., Villegas-Negrete N., Abundiz-Yañez K., Yasbin R.E., Robleto E.A., Pedraza-Reyes M. (2020). Role of Mfd and GreA in *Bacillus subtilis* base excision repair-dependent stationary-phase mutagenesis. J. Bacteriol..

[34] Suárez V.P., Martínez L.E., Leyva-Sánchez H.C., Valenzuela-García L.I., Lara-Martínez R., Jiménez-García L.F., Ramírez-Ramírez N., Obregon-Herrera A., Cuéllar-Cruz M., Robleto E.A. (2021). Transcriptional coupling and repair of 8-OxoG activate a RecA-dependent checkpoint that controls the onset of sporulation in *Bacillus subtilis*. Sci. Rep..

[35] Bagyan I., Noback M., Bron S., Paidhungat M., Setlow P. (1998). Characterization of yhcN, a new forespore-specific gene of Bacillus subtilis. Gene.

[36] Spizizen J. (1958). Transformation of biochemically deficient strains of *Bacillus subtilis* by deoxyribonucleate. Proc. Natl. Acad. Sci. USA.

[37] Neuhoff V., Arold N., Taube D., Ehrhardt W. (1988). Improved staining of proteins in polyacrylamide gels including isoelectric focusing gels with clear background at nanogram sensitivity using coomassie brilliant blue G-250 and R-250. Electrophoresis.

[38] Zuccoli G.S., Martins-de-Souza D., Guest P.C., Rehen S.K., Nascimento J.M. (2017). Combining patient-reprogrammed neural cells and proteomics as a model to study psychiatric disorders. Proteomic Methods Neuropsychiatr. Res..

[39] Ramírez-Flores C.J., Cruz-Mirón R., Mondragón-Castelán M.E., González-Pozos S., Ríos-Castro E., Mondragón-Flores R. (2019). Proteomic and structural characterization of self-assembled vesicles from excretion/secretion products of *Toxoplasma gondii*. J. Proteom..

[40] Geromanos S.J., Hughes C., Golick D., Ciavarini S., Gorenstein M.V., Richardson K., Hoyes J.B., Vissers J.P., Langridge J.I. (2011). Simulating and validating proteomics data and search results. Proteomics.

[41] Valentine S.J., Ewing M.A., Dilger J.M., Glover M.S., Geromanos S., Hughes C., Clemmer D.E. (2011). Using ion mobility data to improve peptide identification: Intrinsic Amino acid size parameters. J. Proteome Res..

[42] Skipp P.J.S., Hughes C., McKenna T., Edwards R., Langridge J., Thomson N.R., Clarke I.N. (2016). Quantitative proteomics of the infectious and replicative forms of *Chlamydia trachomatis*. PLoS ONE.

[43] Engelmann S., Hecker M. (1996). Impaired oxidative stress resistance of *Bacillus subtilis sigB* mutants and the role of *katA* and *katE*. FEMS Microbiol. Lett..

[44] Pybus C., Pedraza-Reyes M., Ross C.A., Martin H., Ona K., Yasbin R.E., Robleto E. (2010). Transcription-associated mutation in *Bacillus subtilis* cells under stress. J. Bacteriol..

[45] Ross C., Pybus C., Pedraza-Reyes M., Sung H.-M., Yasbin R.E., Robleto E. (2006). Novel role of mfd: Effects on stationary-phase mutagenesis *in Bacillus subtilis*. J. Bacteriol..

[46] Martin H.A., Porter K.E., Vallin C., Ermi T., Contreras N., Pedraza-Reyes M., Robleto E.A. (2019). Mfd protects against oxidative stress in Bacillus subtilis independently of its canonical function in DNA repair. BMC Microbiol..

[47] Moszer I., Jones L.M., Moreira S., Fabry C., Danchin A. (2002). SubtiList: The reference database for the Bacillus subtilis genome. Nucleic Acids Res..

[48] Pedreira T., Elfmann C., Stülke J. (2022). The current state of *Subti*Wiki, the database for the model organism *Bacillus subtilis*. Nucleic Acids Res..

[49] Fuangthong M., Herbig A.F., Bsat N., Helmann J.D. (2002). Regulation of the *Bacillus subtilis fur* and *perR* genes by PerR: Not all members of the PerR regulon are peroxide inducible. J. Bacteriol..

[50] Cheo D.L., Bayles K.W., E Yasbin R. (1993). Elucidation of regulatory elements that control damage induction and competence induction of the Bacillus subtilis SOS system. J. Bacteriol..

[51] Au N., Kuester-Schoeck E., Mandava V., Bothwell L.E., Canny S.P., Chachu K., Colavito S.A., Fuller S.N., Groban E.S., Hensley L.A. (2005). Genetic composition of the *Bacillus subtilis* SOS system. J. Bacteriol..

[52] Smaldone G.T., Helmann J.D. (2007). CsoR regulates the copper efflux operon copZA in Bacillus subtilis. Microbiology.

[53] Antelmann H., Engelmann S., Schmid R., Hecker M. (1996). General and oxidative stress responses in Bacillus subtilis: Cloning, expression, and mutation of the alkyl hydroperoxide reductase operon. J. Bacteriol..

[54] Bsat N., Chen L., Helmann J.D. (1996). Mutation of the Bacillus subtilis alkyl hydroperoxide reductase (ahpCF) operon reveals compensatory interactions among hydrogen peroxide stress genes. J. Bacteriol..

[55] Even S., Burguière P., Auger S., Soutourina O., Danchin A., Martin-Verstraete I. (2006). Global control of cysteine metabolism by CymR in *Bacillus subtilis*. J. Bacteriol..

[56] Esquilin-Lebron K., Dubrac S., Barras F., Boyd J.M. (2021). Bacterial approaches for assembling iron-sulfur proteins. mBio.

[57] Gaballa A., Wang T., Ye R.W., Helmann J.D. (2002). Functional analysis of the *Bacillus subtilis* Zur regulon. J. Bacteriol..

[58] Bramkamp M. (2010). The putative *Bacillus subtilis* L,D-transpeptidase YciB is a lipoprotein that localizes to the cell poles in a divisome-dependent manner. Arch. Microbiol..

[59] Micka B., Groch N., Heinemann U., A Marahiel M. (1991). Molecular cloning, nucleotide sequence, and characterization of the Bacillus subtilis gene encoding the DNA-binding protein HBsu. J. Bacteriol..

[60] Karaboja X., Wang X. (2022). HBsu Is Required for the Initiation of DNA Replication in *Bacillus subtilis*. J. Bacteriol..

[61] Price C.W., Fawcett P., Cérémonie H., Su N., Murphy C.K., Youngman P. (2001). Genome-wide analysis of the general stress response in *Bacillus subtilis*. Mol. Microbiol..

[62] Baichoo N., Wang T., Ye R., Helmann J.D. (2002). Global analysis of the *Bacillus subtilis* Fur regulon and the iron starvation stimulon. Mol. Microbiol..

[63] Santos-Escobar F., Gutiérrez-Corona J.F., Pedraza-Reyes M. (2014). Role of *Bacillus subtilis* error prevention oxidized guanine system in counteracting hexavalent chromium-promoted oxidative DNA damage. Appl. Environ. Microbiol..

[64] Faulkner M.J., Ma Z., Fuangthong M., Helmann J.D. (2012). Derepression of the *Bacillus subtilis* PerR peroxide stress response leads to iron deficiency. J. Bacteriol..

[65] Aliashkevich A., Cava F. (2022). LD-transpeptidases: The great unknown among the peptidoglycan cross-linkers. FEBS J..

[66] Bernal-Cabas M., Ayala J.A., Raivio T.L. (2015). The Cpx envelope stress response modifies peptidoglycan cross-linking via the L,D-transpeptidase LdtD and the novel protein YgaU. J. Bacteriol..

[67] Delhaye A., Collet J.-F., Laloux G. (2016). Fine-tuning of the Cpx envelope stress response is required for cell wall homeostasis in *Escherichia coli*. mBio.

[68] Tanous C., Soutourina O., Raynal B., Hullo M.-F., Mervelet P., Gilles A.-M., Noirot P., Danchin A., England P., Martin-Verstraete I. (2008). The CymR regulator in complex with the enzyme CysK controls cysteine metabolism in *Bacillus subtilis*. J. Biol. Chem..

[69] Hullo M.-F., Martin-Verstraete I., Soutourina O. (2010). Complex phenotypes of a mutant inactivated for CymR, the global regulator of cysteine metabolism in Bacillus subtilis. FEMS Microbiol. Lett..

[70] Cha M.-K., Bae Y.-J., Kim K.-J., Park B.-J., Kim I.-H. (2015). Characterization of two alkyl hydroperoxide reductase C homologs alkyl hydroperoxide reductase C_H1 and alkyl hydroperoxide reductase C_H2 in *Bacillus subtilis*. World J. Biol. Chem..

[71] Gout I. (2019). Coenzyme A: A protective thiol in bacterial antioxidant defence. Biochem. Soc. Trans..

[72] Lee J.-W., Soonsanga S., Helmann J.D. (2007). A complex thiolate switch regulates the *Bacillus subtilis* organic peroxide sensor OhrR. Proc. Natl. Acad. Sci. USA.

[73] Höper D., Völker U., Hecker M. (2005). Comprehensive characterization of the contribution of individual SigB-dependent general stress genes to stress resistance of *Bacillus subtilis*. J. Bacteriol..

[74] Yeak K.Y.C., Boekhorst J., Wels M., Abee T., Wells-Bennik M.H.J. (2023). Prediction and validation of novel SigB regulon members in Bacillus subtilis and regulon structure comparison to Bacillales members. BMC Microbiol..

[75] Smits W.K., Dubois J.-Y.F., Bron S., van Dijl J.M., Kuipers O.P. (2005). Tricksy business: Transcriptome analysis reveals the involvement of thioredoxin A in redox homeostasis, oxidative stress, sulfur metabolism, and cellular differentiation in *Bacillus subtilis*. J. Bacteriol..

[76] Garibyan L., Huang T., Kim M., Wolff E., Nguyen A., Nguyen T., Diep A., Hu K., Iverson A., Yang H. (2003). Use of the rpoB gene to determine the specificity of base substitution mutations on the *Escherichia coli* chromosome. DNA Repair.

[77] Price L.B., Vogler A., Pearson T., Busch J.D., Schupp J.M., Keim P. (2003). In vitro selection and characterization of Bacillus anthracis mutants with high-level resistance to ciprofloxacin. Antimicrob. Agents Chemother..

[78] Leehan J.D., Nicholson W.L. (2021). The spectrum of spontaneous rifampin resistance mutations in the *Bacillus subtilis rpoB* gene depends on the growth environment. Appl. Environ. Microbiol..

[79] Leehan J.D., Nicholson W.L. (2022). Environmental dependence of competitive fitness in rifampin-resistant *rpoB* mutants of *Bacillus subtilis*. Appl. Environ. Microbiol..

[80] Patel Y., Soni V., Rhee K.Y., Helmann J.D. (2023). Mutations in *rpoB* that confer rifampicin resistance can alter levels of peptidoglycan precursors and affect β-lactam susceptibility. mBio.

[81] Selby C.P., Lindsey-Boltz L.A., Li W., Sancar A. (2023). Molecular mechanisms of transcription-coupled repair. Annu. Rev. Biochem..

[82] Martin H.A., Sundararajan A., Ermi T.S., Heron R., Gonzales J., Lee K., Anguiano-Mendez D., Schilkey F., Pedraza-Reyes M., Robleto E.A. (2021). Mfd affects global transcription and the physiology of stressed *Bacillus subtilis* cells. Front. Appl. Environ. Microbiol..

