# Supplementary material for: 8-OxoG-Dependent Regulation of Global Protein Responses Leads to Mutagenesis and Stress Survival in Bacillus subtilis
Source: Antioxidants (Basel). 2024 Mar 8;13(3):332. doi: 10.3390/antiox13030332 (PMC10968225; doi:10.3390/antiox13030332)
Supplement: Supplementary file 1 [file antioxidants-13-00332-s001.zip › Suppl Figures.pdf]

SUPPLEMENTARY MATERIAL (Figures)

8-OxoG-Dependent Regulation of Global Protein Responses Leads to Mutagenesis and Stress Survival in *Bacillus subtilis*

Lissett E. Martínez[1], Gerardo Gómez[1], Norma Ramírez[1], Bernardo Franco[1],

Eduardo A. Robleto[2], Mario Pedraza-Reyes[1,*]

[1] Department of Biology, Division of Natural and Exact Sciences, University of Guanajuato, Guanajuato 36050, Mexico

[2] School of Life Sciences, University of Nevada, Las Vegas, NV 89557, USA

* Address correspondence to M. Pedraza-Reyes; pedrama@ugto.mx

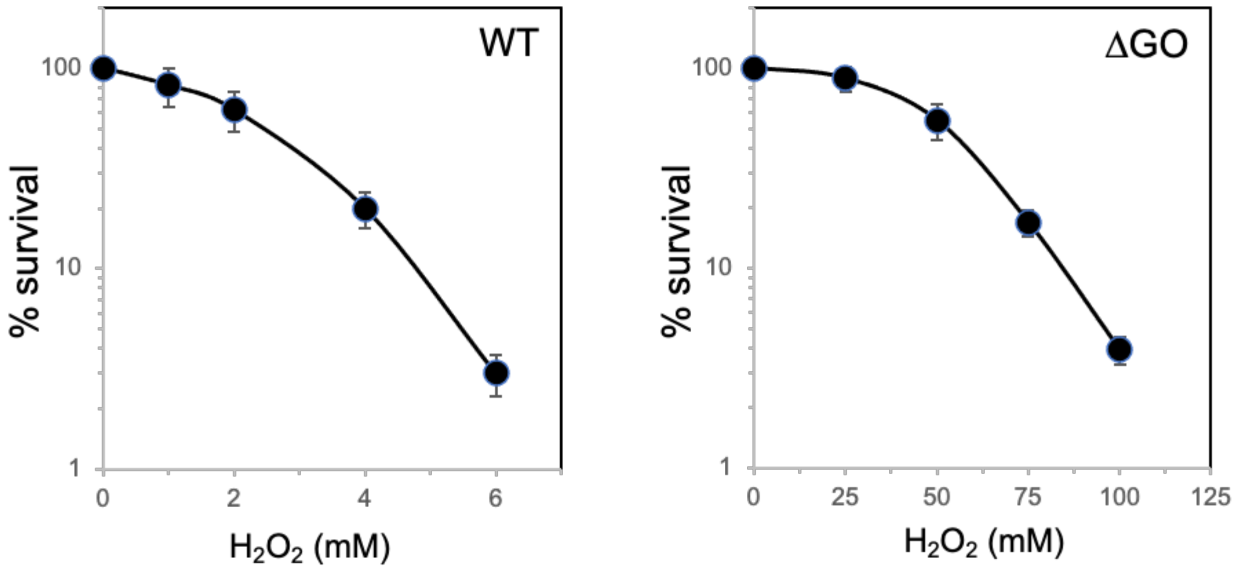

Supplementary Figure S1. Hydrogen peroxide susceptibility of *B. subtilis* strains with distinct genotypes (WT and ΔGO). The indicated strains propagated to an OD_{600} of 1.0 were treated with increasing doses of hydrogen peroxide for 30 min. LD_{90s} values were determined from the curves as described in Materials and Methods. Values represent the average of three independent experiments per triplicate ± standard deviation.

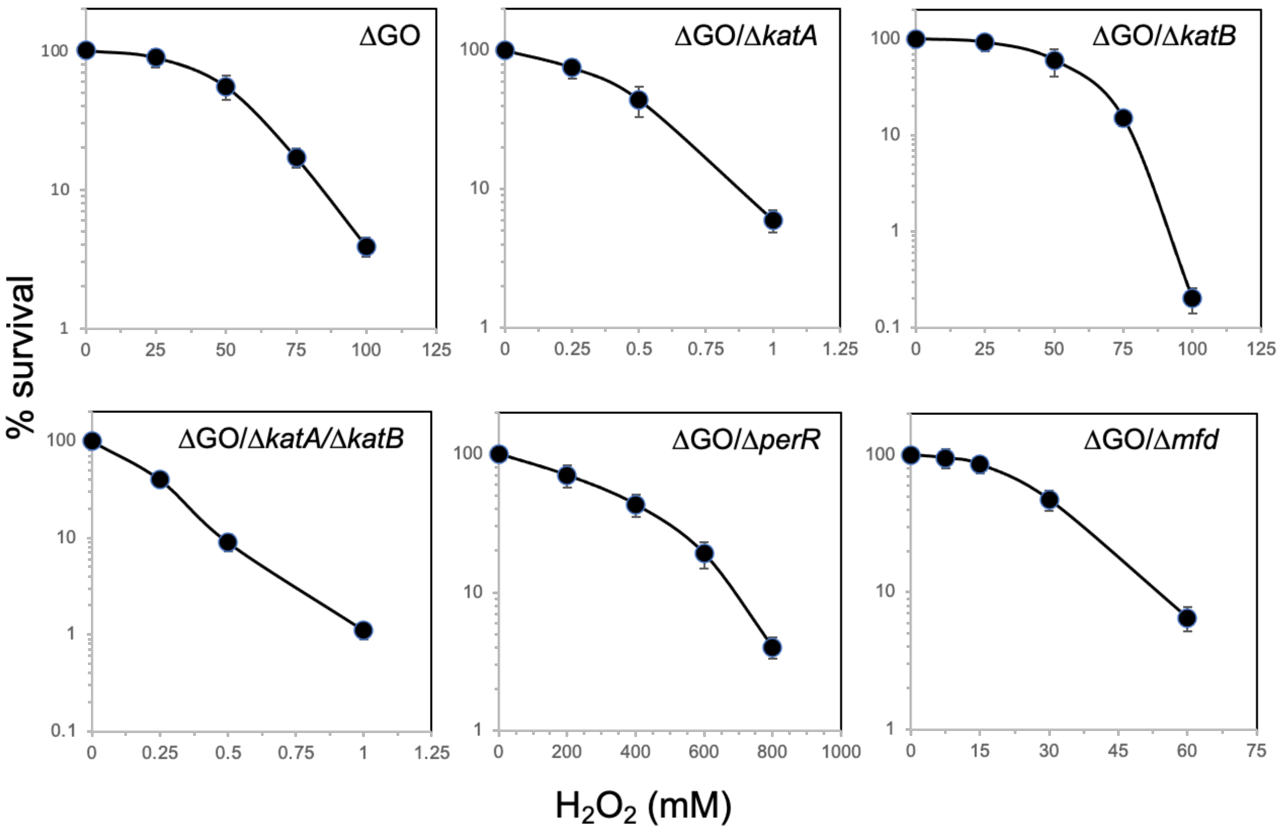

Supplementary Figure S2. Hydrogen peroxide susceptibility of *B. subtilis* strains with distinct genotypes (ΔGO, ΔGO/Δ*katA,* ΔGO/Δ*katB,* ΔGO/Δ*katA*/Δ*katB,* ΔGO/Δ*perR* and ΔGO/Δ*mfd*). The indicated strains propagated to an OD_{600} of 1.0 were treated with increasing doses of hydrogen peroxide for 30 min. LD_{90s} values were determined from the curves as described in Materials and Methods. Values represent the average of three independent experiments per triplicate ± standard deviation.

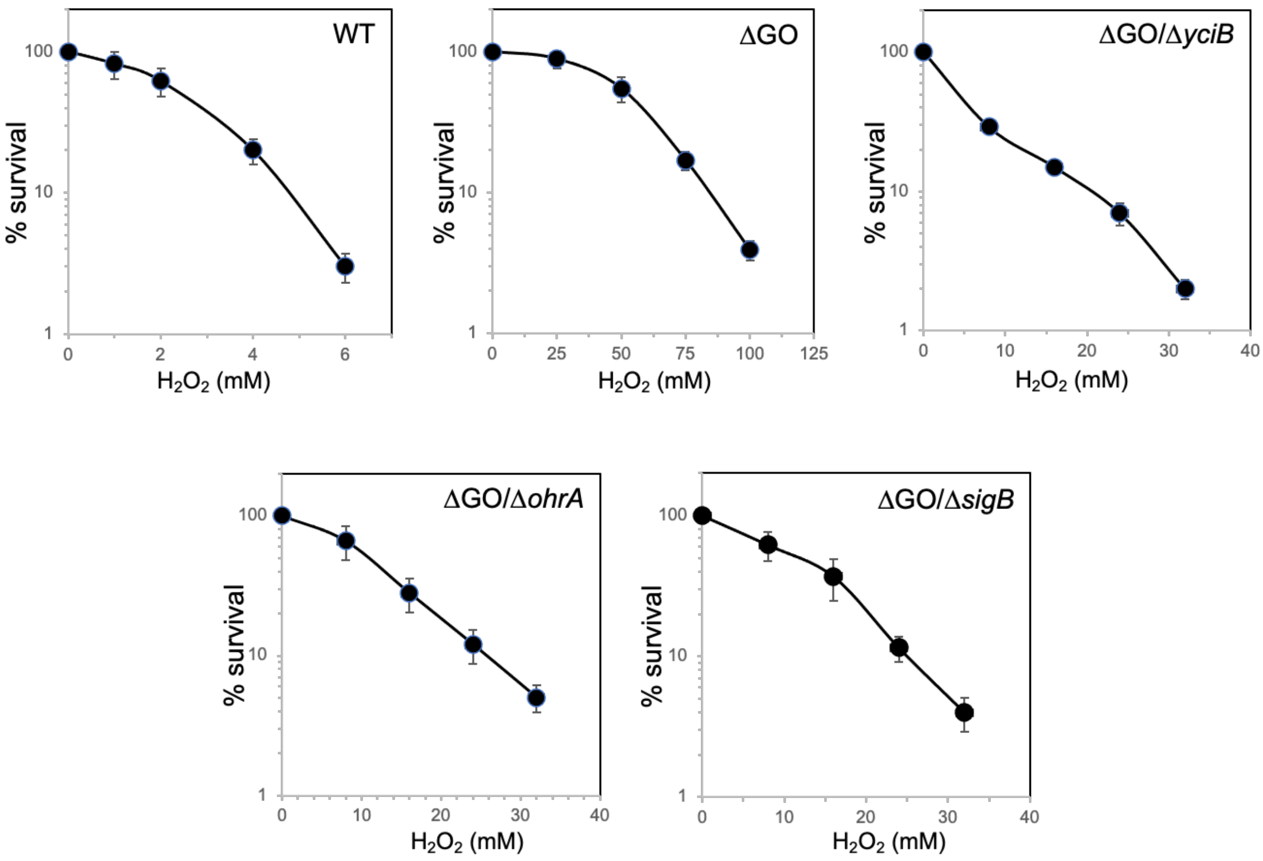

Supplementary Figure S3. Hydrogen peroxide susceptibility of *B. subtilis* strains with distinct genotypes (WT, ΔGO, ΔGO/Δ*yciB*, ΔGO/Δ*ohrA* and ΔGO/Δ*sigB*). The indicated strains propagated to an OD_{600} of 1.0 were treated with increasing doses of hydrogen peroxide for 30 min. LD_{90s} values were determined from the curves as described in Materials and Methods. Values represent the average of three independent experiments per triplicate ± standard deviation.

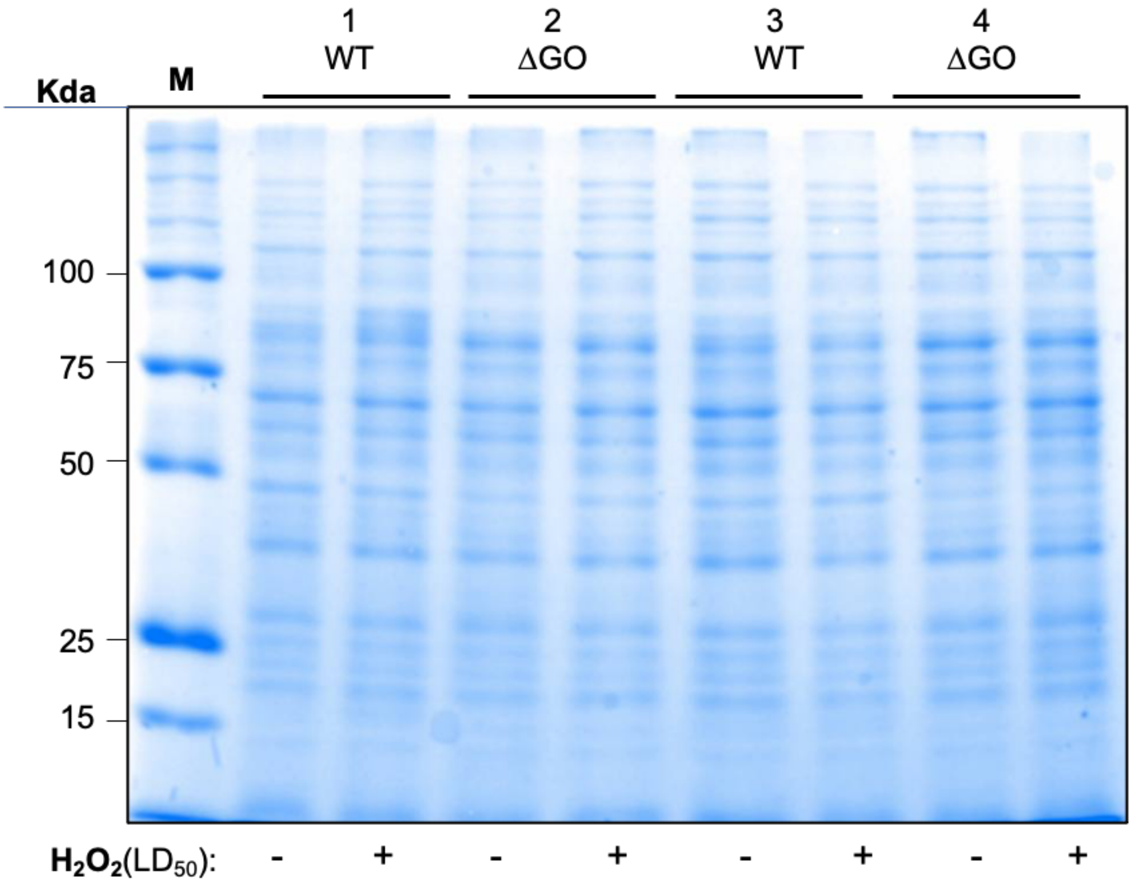

Supplementary Figure S4. SDS-PAGE analysis of protein cell extracts from strains *B. subtilis* WT and ΔGO. Cell extracts (8 mg) obtained from indepent cultures of *B. subtilis* strains WT (1, 3) and ΔGO (2, 4), untreated or treated with a LD_{90} of H_2O_2 were separated in a 10% polyacrylamide gel that was stainned with Coomassie R- 250 blue dye. **M**: Protein markers.

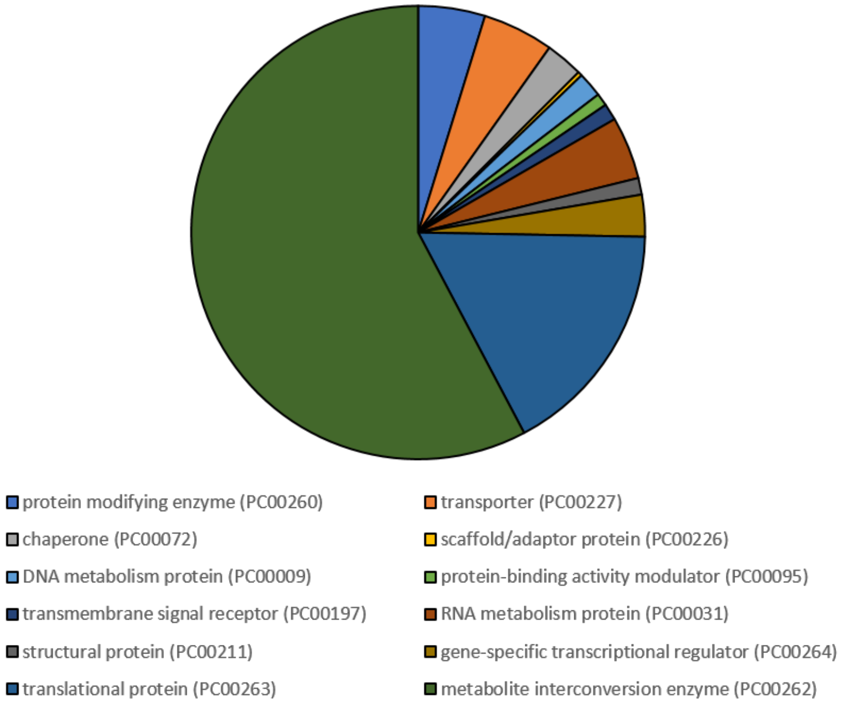

Supplementary Figure S5. Functional distribution of differentially expressed class proteins between the WT and ΔGO *B. subtilis* strains subjected to hydrogen peroxide stress. PANTHER classification analysis was conducted on the hits found by proteomic analysis.

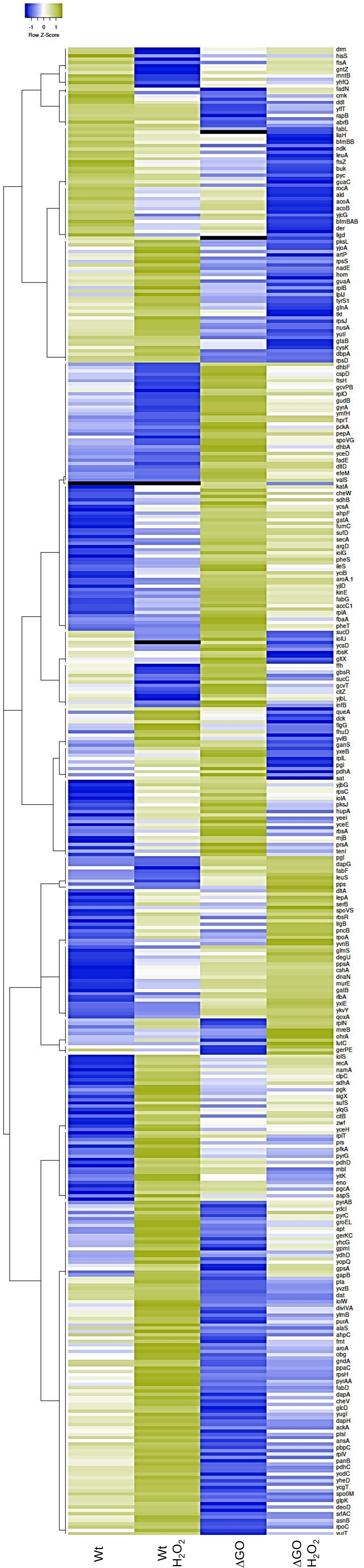

-1 0 1
Row Z-Score
drm
hisS
ftsA
gntZ
mntB
yhfQ
fadN
cmk
ddl
yflT
rapB
abrB
fabL
liaH
bfmBB
ndk
leuA
ftsZ
buk
pyc
guaC
rocA
ald
acoA
acoB
yjcG
bfmBAB
der
ligd
pksL
yjoA
artP
rpsS
nadE
hom
guaA
rplB
lplJ
tyrS1
glnA
tkt
rpsJ
nusA
yutI
gtaB
cysK
dbpA
rpsD
dhbF
cspD
ftsH
gcvPB
rplO
gudB
gyrA
ymfH
hprT
pckA
pepA
spoVG
dhbA
yceD
fadE
dltD
efeM
valS
katA
cheW
sdhB
ycsA
ahpF
gatA
fumC
sufD
secA
argD
iolG
pheS
ileS
yciB
aroA.1
yjlD
kinE
fabG
accC1
rplA
fbaA
pheT
sucD
iolU
ycsD
rbsK
gltX
ffh
gbsR
sucC
gcvT
citZ
yjbL
infB
queA
dck
flgG
fhuD
yvlB
ganS
yxeB
rplL
pgi
pdhA
sat
yjbG
rpsC
iolA
pksJ
hupA
yeeI
yceE
rbsA
rnjB
prsA
tenI
pgl
dapG
fabF
leuS
pps
dltA
lepA
serB
spoVS
rbsR
lrgB
pncB
rpoA
yvnB
glmS
degU
ppsA
cshA
dnaN
murE
gatB
rlbA
yxiE
ykvY
qoxA
rplN
mreB
ohrA
lutC
gerPE
iolS
recA
namA
clpC
sdhA
pgk
sigX
sufS
ylqG
citB
zwf
yceH
rplT
prs
pfkA
pyrG
pdhD
mbl
yitK
eno
pgcA
aspS
pyrAB
ydcI
pyrC
groEL
apt
gerKC
yhcG
gpmI
ydhD
yopQ
gpsA
gapB
pta
yvzB
dat
iolW
divIVA
ylmB
purA
alaS
ahpC
fmt
aroA
obg
gndA
ppaC
rpsH
pyrAA
fabD
cheV
glcD
yugI
dapH
ackA
ptsI
ansA
pbpC
rplV
panB
pdhC
yodC
yheD
ycgT
spo0M
glpK
deoD
srfAC
asnB
rpoC
Wt
Wt H2O2
ΔGO
ΔGO H2O2

Supplementary Figure S6. Complete proteome differences between strains *B. subtilis* WT and ∆GO. Heatmap analysis using Log_2 values of the abundance of each protein. Each target was evaluated for complete linkage and Spearman Rank Correlation.
