# Supplementary material for: 8-OxoG-Dependent Regulation of Global Protein Responses Leads to Mutagenesis and Stress Survival in Bacillus subtilis
Source: Antioxidants (Basel). 2024 Mar 8;13(3):332. doi: 10.3390/antiox13030332 (PMC10968225; doi:10.3390/antiox13030332)
Supplement: Supplementary file 1 [file antioxidants-13-00332-s001.zip › Supplem Data Fold Change Values of B. subtilis WT and GO strains.pdf]

NAME	WT	WT H2O2	GO
oppA	66.2844876	31.5539129	116.193409
rocG	11.1012873	1.1187159	4.83842806
dhbF	8.51313401	5.70110876	19.0240959
pta	63.5932037	76.9351772	49.4212872
rplT	76.7220528	94.2505718	91.2257509
rocA	44.3248163	7.93084664	18.5054246
serA	9.94227206	8.30781045	10.3427878
yxeB	38.2419084	43.867067	52.5925015
ald	128.957536	109.243062	119.508883
yqiG	3.003708	6.68311727	1.79025741
yumC	22.9555737	27.2002958	33.1163224
coaE	0.66947898	0.25978931	6.39219811
katA	5.21294656	87.8612257	227.715764
ffh	2.34984793	0.23210245	6.83356212
gbsR	3.13059856	0.93450198	6.55785951
pyrAB	85.128279	135.42923	83.3004553
rplW	37.1197864	45.7605477	43.8238054
rocD	38.9702137	16.3093242	23.539327
glmS	1.31572478	3.07872553	4.78467965
rplQ	117.739859	147.923922	130.860474
murF	5.19378616	6.46590828	5.72504927
queA	32.822251	38.51642	35.0571777
acoA	36.1474198	9.00278545	26.1140184
aspB	25.2314043	28.1712139	17.1823148
iolS	26.3595547	43.7646773	33.7526234
putC	59.4500589	22.6703936	57.4458492
cheW	12.3541715	12.9303492	15.835926
feuA	2.83825476	1.09460943	0
dhbB	199.4802	221.869362	204.918263
recA	2.72141585	10.8921683	5.85721336
fabL	5.4968116	3.88993573	0.84017868
prs	28.1026314	34.4140377	28.7573479
yvfG	4.59346464	5.71675851	2.98862169
pksL	11.9183091	14.187507	9.62621179
kbl	23.2147537	16.8034051	38.2634972
tpx	111.631733	153.502599	133.010419
acoB	83.401836	30.22378	74.3446406
odhB	90.0460212	101.118463	126.109261
rocF	50.6220331	14.3889944	22.830865
hisA	17.3922563	22.1044703	19.1120285
pfkA	47.821115	60.7368562	51.3582347
pyrF	157.823385	202.203105	49.3766625

salA	20.0282226	29.1176584	27.3457017
yjoA	10.9287602	17.6650553	10.1158038
yvzB	36.3317319	40.639148	9.61404436
acoC	21.7238276	16.9819966	22.9397357
hag	639.352407	736.043146	255.91983
dat	116.197536	143.909046	49.4802482
dltA	8.40294435	13.0409665	10.2804973
ydcI	5.51316763	7.05964925	4.84260734
rpoB	59.5407052	58.5721921	68.7181293
yjbG	21.6043082	25.8633641	26.1448814
hemL	11.7496127	13.472263	23.2932889
cspD	31.334523	25.9331763	51.2649367
wprA	9.3117596	13.717352	14.2723825
sdhB	4.60081065	5.81671899	8.50682429
lepA	6.66036842	7.50931647	7.23537608
yjcG	18.8069018	15.2423893	18.5013206
rplE	103.937205	121.490018	100.517917
yopA	60.5259549	75.8480733	23.3389942
nadC	20.6023314	18.8368857	16.7451657
odhA	105.199661	110.633096	138.350998
liaH	13.6162681	11.2718827	9.52015644
sucC	148.007343	122.510741	171.131291
clpE	2.63637014	3.30491538	3.58480287
ycsA	3.4760094	4.76717376	6.46053253
yvgN	34.7002227	48.6863808	54.6155222
clpP	23.8852904	31.9069623	26.1414796
ahpF	19.5535017	39.2488391	169.196237
rpsC	162.567	180.302667	182.076138
xkdC	2.80771307	3.70613678	4.6338842
pyrG	24.6964436	28.0135601	25.2081233
mntB	1.05809038	0.48929988	0.74067481
pyrB	9.24719994	15.451222	8.57392662
acoL	23.7162715	14.4917152	21.9185932
yvcT	28.6694223	32.7465513	31.8930503
gabD	9.31497541	12.9374739	11.9796881
iolA	31.1450716	55.0554494	61.3264684
iolW	21.6626983	24.826288	19.0570635
ydjF	28.2896415	31.620537	28.7022458
atpG	21.3602707	9.32688679	23.5289777
rplD	84.6304158	111.935745	76.9078033
gcvT	7.3129947	5.80437543	12.4871562
sucD	55.7774093	47.2642752	58.7489302
yqeY	13.5365818	8.8694452	11.4046761

divIVA	41.1168896	49.1410904	36.5935838
yvyD	8.17653139	9.96299899	6.19052328
ylmB	6.26833826	7.16020447	5.4755891
pdhD	174.469881	188.993321	185.805279
iolU	1.28516281	0.80416978	2.14860672
prfA	5.88828795	7.95946964	2.51143551
malS	3.17055616	1.79558999	3.1536737
namA	2.25110988	7.04454581	5.38963002
drm	11.6330529	4.82809306	9.39095471
metK	33.2628578	37.4558675	34.96301
purA	6.9706755	8.50821502	4.71286649
yqiT	26.1930817	24.30226	24.078636
yfkI	15.0417219	18.8713792	14.3917403
yknY	4.59816317	10.8444748	8.93364669
sufC	36.6467991	53.1183962	77.1509435
bfmBAB	19.2294022	15.3254085	15.3302644
yhaM	36.7715968	31.9673339	34.4779383
clpC	36.7309412	43.8046739	41.1094169
serB	7.50919622	9.30350769	8.1275231
gerPE	5.22182885	2.85181927	1.69005447
artP	15.7284213	17.2375778	15.0037951
rplU	115.81824	129.076161	135.436004
alaS	104.385551	154.810919	9.72981084
hisS	7.2763656	5.70662398	5.95932734
adk	20.7390675	24.9030442	19.648668
gatA	33.4013862	37.5321876	40.5113483
rplF	51.9590887	63.8876303	56.2354568
sdhA	4.73954524	6.79431657	5.69426846
pyrC	5.47631347	8.93102095	4.22165529
ahpC	250.286535	478.278169	0.01977544
bfmBC	24.9023375	32.4905438	24.7989686
icd	22.1843181	13.7671279	20.3529587
copZ	0.99927829	2.16849801	1.98527747
pksJ	37.9112088	40.8135601	44.1113099
iolH	1.49440427	3.04649705	3.32016856
smc	3.04909329	4.94652394	9.36561865
bfmBB	37.7414373	33.2943151	32.5164216
pyrH	14.8366852	16.6640094	17.3051839
rplM	18.6952538	22.5918838	19.7079347
ylbN	4.06152034	8.67918359	1.94666406
fumC	36.9047472	55.3972501	68.0144296
groEL	89.7967363	127.875147	89.2956937
dck	7.09070139	9.79847036	7.12488996

degU	1.70794206	2.45201035	3.631446
yxlF	4.77656627	7.25474883	23.5554157
dhaS	49.7631386	39.7433424	75.4537116
spoVS	21.1125657	24.8379005	24.7477769
pgk	116.200237	146.700257	119.788063
ftsH	9.72386677	7.8785644	16.6839443
nadK2	9.37290893	7.65899424	11.9504816
cmk	3.75810915	3.21970102	3.00036973
pyk	62.6480705	75.5922041	59.5882922
gcvPB	12.9079015	10.7680929	16.4107966
rpsB	62.5830004	83.8808809	66.5318182
mbl	29.3371734	38.8518213	36.7896037
fmt	4.54714459	4.95109937	2.87321139
pdhB	238.640964	298.508662	222.596371
sufD	25.5752753	30.9469546	61.2326156
ypfD	77.4546062	65.7017344	99.6262715
hupA	303.465688	359.108447	401.52926
aroA	32.2950111	40.3936761	27.4497369
fusA	115.909225	148.154765	148.828079
gyrB	53.4477838	66.2065383	43.014744
iolC	18.8353188	28.5724921	38.3745271
ychF	24.6693618	28.5325645	27.6922766
apt	18.1203686	23.4746067	17.7468005
tpiA	31.9141964	39.3529894	30.4095001
citZ	40.6567098	34.3813938	48.0831211
secA	4.01691082	4.6191225	8.12002531
grpE	51.4840562	56.6998427	60.2760529
yqjE	4.54720099	4.80852348	4.68117591
obg	7.56293661	10.2601338	6.08059543
pnp	28.0793037	33.2096383	27.9195034
serS	24.2186566	15.4503337	21.3463962
yhfK	8.1009218	11.9626337	9.77537171
thiN	1.4248556	0	1.54508835
ppsA	2.50123454	3.96516475	5.33585923
rpmC	41.7324995	50.3613918	36.283849
yugJ	10.769641	18.4071699	21.3943118
cshA	51.7164367	61.3052083	66.9584723
yitK	9.3542891	12.6531919	10.4586856
fliY	16.243924	16.8976148	34.7023991
rpsS	37.0815524	47.1202609	36.2737382
gerKC	3.12461662	6.10320734	2.62600447
der	2.69816893	2.09948204	2.58648814
ddl	19.0357651	18.7045567	18.0893086

yeeI	14.3379702	15.9605893	16.7135564
gndA	16.4194929	17.0204603	13.3564123
dhbE	42.8135079	48.4457597	76.334652
ykwC	6.86304682	6.22644686	1.00083126
yjjA	9.28053633	6.1336621	11.0286055
argD	7.52162157	14.4447502	21.0183308
sigX	23.9160113	27.9862133	25.5579838
yhfQ	6.35342268	4.96400938	5.5135135
guaB	69.1241553	77.3582333	44.8944348
nadE	36.1174884	44.7829672	29.6074529
accD	8.16582159	6.30041465	8.94806897
yheA	20.0555806	24.7157728	19.7128225
hom	16.3235202	18.8129735	13.5544355
bipA	22.4900817	25.7474174	21.061017
purL	33.5131515	35.4117645	36.1743458
cymR	1.32081054	3.69686374	27.8149044
nusG	6.2554518	8.96068994	6.78527854
yfmC	13.0778169	14.0698785	12.7591532
yjbL	22.8329489	16.0546356	23.7643034
metG	20.6382236	26.5223676	22.3284928
rplO	6.17956807	5.49481915	7.69907209
clpX	23.9038463	20.7347839	29.7271402
eno	247.302908	277.56723	271.62964
rbsR	29.671579	33.9456771	30.114307
ytsJ	13.7225536	17.5117598	1.39866082
iolG	18.7066074	21.3695113	27.7804725
cysS	7.33283743	8.46149008	8.25702953
yhcG	3.26508363	4.2997161	3.06582614
floA	6.37617508	8.20009925	9.16985693
ppaC	50.5777082	64.112072	44.4155972
yceE	19.295924	25.3852233	28.2257001
dnaN	12.5850391	14.6167083	14.9705339
asnS	12.2065592	14.2563766	9.22289008
rpsH	38.9856736	49.5452581	33.5914066
sufS	10.793507	17.656646	16.040935
tuf	476.540965	516.140298	541.92997
flgG	16.5416005	24.4641317	16.698344
fabI	33.6574566	36.5461936	36.2062904
yoxD	11.6684043	15.26686	9.15932597
pyrAA	8.80281647	11.6763782	7.35102164
gudB	24.4406553	19.6736867	38.5445451
fliG	4.94588656	3.79257144	9.36887703
iolE	14.0512832	24.1699356	29.2113474

putB	11.5044917	8.63947981	9.10279181
licH	96.7573985	88.6966978	45.4700509
pgcA	23.0348767	26.7442178	26.1959267
tig	229.482025	268.961905	264.33005
ytsP	5.76412153	8.29208194	4.91457143
fabD	13.0718979	14.842386	8.62675376
pgl	3.95135508	3.29065086	13.6632382
ftsA	11.7703811	10.7822707	10.9395233
ndk	15.8681711	15.2376136	14.9207751
gyrA	7.89036857	6.36197415	10.3223019
pheS	8.0842159	9.79009009	14.6674763
frr	64.3013308	74.2907554	55.7442667
guaA	110.973872	125.575123	84.2148165
rpsG	208.753305	251.56346	212.528245
dnaK	54.2050748	59.1697128	75.5750584
dapA	7.95401351	8.79196775	6.00177278
lrgB	1.7360137	3.5252901	2.10367906
ytpR	8.30918687	14.5311787	4.83881896
thrS	9.37370702	11.1619932	7.86337866
cheV	10.0242358	12.1503095	7.78950983
yfkO	3.07256852	4.51363814	1.70794799
ileS	6.6880079	7.08598213	9.58670523
ampS	20.1051501	22.3212982	28.6960599
rplB	141.78549	165.494639	141.191655
glcD	7.73594087	8.85359932	5.3807821
malR	2.64374983	2.37548748	2.34092074
yfiY	18.7209888	19.7394143	29.318287
yciB	0.54369991	3.02916993	15.593276
iolI	22.1162611	34.9706158	51.5170041
murAB	6.22230137	7.92397727	1.03449964
yugI	16.8401534	22.5252799	12.5740681
fhuD	23.7604379	30.5942603	28.3447614
glxK	11.6685381	14.6135512	7.0935526
punA	20.6766463	27.2429823	18.3495985
tcyK	21.6821323	26.0436206	21.9264359
aroC	6.39575816	7.37052784	4.22496571
dapH	6.90919743	8.18871	5.3634365
rbsA	23.2037635	25.2256317	31.4112633
yerC	1.54561434	1.07713924	1.42926519
aroA	1.2844365	1.6528698	5.45944699
ylqG	12.8453992	17.9558311	14.9454523
lplJ	6.11602888	10.1263412	5.92304148
menB	45.3761032	41.8240697	51.9256972

rplN	133.413171	140.138152	130.410223
gpmI	5.96586246	7.71328326	4.4884521
pdxS	27.218608	38.9097769	9.74934038
iolJ	11.3504012	15.9855997	14.8430902
aspS	19.2607678	22.8159699	20.2443728
ackA	92.6907331	106.782808	82.3988165
srfAB	96.9524155	107.759018	106.019572
yqbO	4.51307636	5.60930715	7.59132994
yfmS	47.572336	56.900522	32.3572451
tyrS1	12.8424343	13.9396195	11.1241125
ysdC	4.79824055	5.62326936	4.4128916
rplL	85.962777	88.9343328	93.5427604
mtrB	1.95749475	2.65134528	2.55932576
ycsD	1.35039964	0.65888549	2.12707352
nfrA1	3.62366392	5.26345468	2.2494566
ymfH	6.3506467	5.77026824	8.8440831
sufB	26.7066981	31.4529429	62.1451815
mntR	2.90775758	4.19406772	2.261144
groES	12.1856687	18.4523227	12.010428
ptsI	47.3694585	57.0277221	37.0837051
gmk	9.32513356	10.8932842	5.1332285
yrkQ	8.0727928	6.75815136	4.5888319
yjlD	24.4818772	27.5605502	29.9848146
ansA	9.63640216	13.467229	6.45055206
dapG	2.27113037	2.04928241	4.97707754
hemB	0.97143446	0.78629748	3.18768311
iscS2	9.28397927	16.8104452	0.47796997
murE	2.2485864	3.34703963	5.28112264
tgt	1.72794998	1.99617467	2.33992284
hprT	6.76002607	6.12035382	8.96467096
citB	49.2642107	58.521376	50.496734
ptsG	3.26889936	4.60180348	3.96037305
pbpC	8.89301953	10.1381421	6.14340432
ADH1	88.1653098	102.276826	90.7554625
argS	1.73333838	1.31116302	1.81526097
glnA	41.7904669	47.1526726	34.2452585
ydhD	70.8175253	86.1474247	69.9603246
metC	32.2785244	39.0443796	25.0015569
serC	19.2553497	18.4245731	21.6541613
ypgR	2.4278712	4.12426801	1.98023682
yopQ	3.96348513	5.56038288	3.6799178
wapA	22.1313821	22.5135979	28.0785967
mdh	205.352464	230.328542	241.633642

zwf	25.3847259	29.3058371	27.9486975
rpsK	47.0895059	49.443513	53.6283333
yflT	13.3239711	12.4776144	1.89285363
rplV	58.0997349	61.2711712	50.7956865
hemAT	16.4523165	22.1988177	11.6551105
rnjB	5.07752206	5.87978244	6.09878821
panB	2.03086719	2.80979667	1.159723
rbsB	28.8558494	25.3940764	38.6631176
ligd	3.16494447	2.11209373	2.4928247
ilvC	7.7754642	11.5614103	5.11604672
kinE	1.95167873	2.45177369	5.14956505
ykuJ	15.1347014	15.8352504	15.4419652
murD	6.72998917	6.4555236	7.74421394
srfAA	24.7356283	26.0381422	37.0143857
fabF	0.84937233	0.68039479	2.09741375
codY	34.8031162	35.8022483	34.7973555
pncB	8.29372508	9.54685785	8.41924777
gatB	6.9892537	7.89707315	7.96701421
leuA	19.9999107	18.1067992	17.4148986
yclQ	4.04169756	3.65139576	2.44607612
murC	13.9414252	11.2911686	13.3767132
tkt	234.880652	239.52092	220.714321
yvlB	7.0914687	7.89048339	7.10995955
fabG	25.482327	28.9248174	37.4880279
ftsZ	18.3567787	15.5956855	15.0188672
yukE	13.2698037	14.2548358	93.9291172
tsf	170.035899	192.656826	155.269944
rpsJ	158.895931	180.151009	136.661043
pdhC	30.4666035	35.7602255	21.7134415
iolD	21.2231151	23.9123228	18.2567695
gapA	269.738011	287.882599	234.188617
rlbA	3.92523449	4.16341492	4.64839119
yodC	3.32219277	4.17133709	1.51469805
pckA	66.1763104	63.9695647	78.9864778
trxA	1.17689665	1.97914784	2.34699762
cspB	82.071528	88.3826934	87.8119115
pgi	191.757303	201.950239	203.81505
rnjA	5.16093864	5.78998782	4.4380025
ydjI	4.0144446	3.69280524	5.8164768
rplS	43.4649047	45.7542696	50.7132246
ykvZ	0.85835407	2.47304525	0.1618982
accC1	11.5905668	12.7459975	15.8902063
yheD	4.36324864	5.85645542	2.22461324

infB	131.498045	115.762942	146.097377
pepT	12.3795736	13.0959206	13.5428883
lipA	4.41275451	7.43057038	1.39512619
engB	22.1390993	15.7485343	13.6550804
ycgT	4.09011569	5.6929435	1.85959984
ponA	11.4857106	13.4014676	12.1886006
rpoA	27.4668564	27.8782564	27.5975952
pyrE	10.0702096	11.8415992	9.93925939
tcyA	9.90433789	10.5889168	8.09334621
glyA	10.5653142	9.65316396	15.3431612
pepA	1.83291489	1.68559197	3.57323912
fliD	6.48345531	5.17954629	7.51613465
gpsA	3.95583453	4.30426681	3.30987304
atpC	3.08249593	3.49189833	5.16757321
atpD	78.1238587	84.6479721	105.393014
spo0M	24.0381771	25.3085757	8.69520285
yfjO	33.1730793	34.2475692	30.5417277
yxiE	1.34377043	1.4370305	2.54102491
gntZ	2.86566605	2.61944359	2.78496418
fabHA	0.6124704	1.12612633	0.61043178
rbsK	47.6560682	46.0181367	49.5804889
glpK	10.6356993	11.2764755	9.47922343
ganS	3.76525904	5.52156749	5.04545308
melA	8.37326786	8.22878845	5.0297926
glcR	0.92121806	1.16454223	0.31791738
yceH	2.1364166	2.22657809	2.15958
infC	13.1940871	13.758138	18.4142242
minD	0.33575426	0.49838097	0.479587
rapB	4.85471534	4.81176072	2.23219238
prsA	49.3526503	52.7821744	75.5358262
yvnB	0.17603778	0.25549379	0.19813055
ykuP	43.1917928	39.9348585	48.4272691
buk	10.1189327	8.95175506	7.10216516
spoVG	10.8464617	9.43103551	16.15855
ykvY	3.44079879	3.64757689	5.03882972
clpY	6.51907264	6.61420542	7.04245173
mleA	7.74017345	8.11234473	8.30667217
sdaAA	4.9012442	4.77898684	0.88354377
deoD	9.86138664	10.1320069	7.46955427
yokG	14.8193044	12.1963115	10.9813967
abrB	19.2007434	16.2301624	12.5003048
rapC	1.04540089	0.99935901	0
pyc	28.1034495	26.831132	22.0049308

cadA	4.76198159	3.30746373	12.9058858
pdhA	34.715087	35.5784625	35.6968148
glpD	24.0117403	24.997462	21.6645104
tenI	11.904709	12.1729209	14.1252385
dhbA	30.8611477	29.9569553	51.677667
rplA	61.5139744	63.6793749	66.9509753
nusA	21.2455785	22.0523388	19.9795863
yvfR	26.8965843	32.3129745	20.013482
yfhC	83.3275445	81.2877002	69.9122245
yutI	15.8753985	16.6314392	14.3900076
mreB	11.5460156	11.8453621	11.399873
gltX	29.5833158	29.2537126	31.6549516
ytkP	1.69703003	1.75323053	1.69598272
dhbC	33.2462129	32.4266819	45.7243352
atpA	136.875616	141.466696	199.388772
yceD	21.1271699	18.9355044	34.9213205
leuS	18.8095088	18.5262949	19.5265597
fbaA	182.547168	184.834614	208.240273
guaC	8.00739663	5.58987366	5.50249231
ppiB	14.8601707	14.3149707	17.9384918
yfmT	36.519178	37.0122003	18.4964166
trmFO	2.79303507	2.61792189	1.36239289
srfAC	17.2014633	18.6478309	9.28793324
greA	10.7146215	10.3484251	12.0401418
rpsF	66.0038688	64.9882029	65.8696623
rpsE	61.3368866	63.5014795	68.2128786
sat	21.4171481	21.596267	21.9767701
ylbA	1.78943369	2.04624054	1.53939892
proS	12.4451168	12.3896348	7.07293141
asnB	10.6596885	15.6459192	5.59383164
rsmA	4.38980639	4.40706851	5.45589441
rpmA	24.3069578	25.6813454	18.4580983
ohrA	0.42619696	0.45043588	0.23243869
fadE	4.02067443	3.90023418	5.73169097
gtaB	33.5536911	33.895008	31.1048686
rpoC	120.43382	121.425181	104.850393
rbsD	7.68111428	7.71962664	6.90563293
yobI	21.0629461	20.1550172	22.7716281
cysK	94.0164038	97.8465595	91.8556527
cheA	17.3158305	15.4049872	18.8012378
dltD	4.73361411	4.54365755	6.27700149
rplC	76.0467335	77.6408085	74.1502709
tuaD	1.73438915	1.60780931	1.08913118

glmM	8.26049432	8.19730851	10.0474264
rplK	208.550552	215.548632	173.701559
yurT	2.26169508	2.33309389	0.50336509
efeM	71.8972271	70.8142795	81.2210033
pps	7.92167775	7.84873062	10.6573435
pheT	15.3462367	15.4985295	23.4306743
dbpA	14.6204741	14.9881883	14.039421
gapB	30.4551634	32.5565621	30.0016979
bdhA	70.3552131	71.0659763	78.1955171
yueB	7.28135286	7.07322401	8.91055924
qoxA	3.42636682	4.05320089	5.77167943
ykoW	1.20510996	1.7662412	0.87052275
yerO	2.90562559	3.12648172	1.28423759
rpsD	18.5302935	19.1701413	13.6528812
speB	0.77498666	0.74199929	0.37925586
valS	50.2370204	49.9605355	68.2295497
tal	10.7679892	12.6517315	14.0090742
fadN	9.63897327	7.3542171	3.72166607
lutC	3.26608238	3.27026197	3.16805639
sigF	0	0	2.20840004

GOH2O2
57.9155637
0.19322503
10.6124288
53.643534
82.010021
2.02836387
8.09607804
35.9356163
89.3285784
4.75200675
27.3791259
6.02685708
161.066423
2.29139197
1.88921546
108.731605
43.7269299
10.1601807
6.84321342
121.18248
6.33866974
32.0884621
2.3704689
14.45035
39.2359819
7.69211161
15.5311635
0.11548668
143.040322
10.0710353
0.51807481
28.6733691
3.69424804
9.06657649
24.1346248
132.296029
13.5282573
103.616125
3.7403667
21.0219034
50.2588913
61.9249826

30.3364823
7.1130761
12.186043
17.2053351
314.153898
53.4668633
17.8342588
5.60902415
69.2067039
23.6221195
23.7668531
33.4845545
13.2215681
8.14087359
8.22200494
14.7888053
100.37005
24.5922884
18.2410939
116.204481
6.50609649
127.771204
3.2659202
5.2905227
50.210954
25.1209846
137.360393
177.36678
3.62719091
24.9189744
0.70498554
15.0301507
10.6070005
34.6413051
11.3392598
51.2386298
19.5750275
25.6820665
16.7526251
80.7886538
6.82556681
45.6507268
12.7878116

40.3703747
7.56940561
5.65701755
179.465052
0.79087442
3.58433882
3.07899539
5.6427241
10.737212
36.180418
6.1472898
17.7659514
14.6045783
8.66371093
73.8042777
12.6914115
29.0043336
41.1178582
9.43311693
5.2503954
12.9033424
123.446576
10.6050774
5.96571172
20.2723157
37.7970967
59.038317
6.48180465
6.59142298
0.05736371
28.4303036
16.4124352
18.2989826
39.4900074
7.16487036
8.76530073
26.7663519
15.6303994
20.1235292
3.7761856
58.031982
95.1289408
5.62657414

4.40221888
20.4134211
58.3012688
27.1785953
120.541198
13.5683794
9.83832343
3.12159258
63.2372294
13.0768191
70.5892404
29.7526254
3.62838664
236.295301
58.8646455
75.8124697
306.247023
29.6346606
143.83051
42.7355874
32.9043982
25.2557485
20.6568321
31.1371243
39.3107528
7.17803934
63.3865014
3.60801356
7.29085363
29.6020628
22.434471
12.0877677
0.11285713
5.61682114
38.4915765
22.1023111
68.0482237
9.68092244
26.6066713
35.385305
3.40425079
1.6972918
18.1144115

14.564102
13.9855196
44.0354393
1.95745098
9.0947513
17.3116851
26.393097
5.36985284
40.0754953
29.2091036
8.20434284
18.5252251
12.2185657
24.0551367
32.2463959
24.0035309
7.53823087
10.2270682
22.7514594
20.8129021
7.44979225
24.9165488
255.666538
36.05085
2.40973351
23.5861118
7.87701397
3.44120667
9.5698678
46.4712164
24.9433459
15.7883746
9.72438278
34.1493791
17.2621125
477.275987
14.6920134
36.7899351
9.32420967
8.20994155
28.3746596
6.94091582
26.8216051

4.14923584
42.9558516
24.078419
258.961082
5.30504676
9.36043706
19.1639327
11.553843
11.2975751
8.67245914
14.2401641
58.2810361
83.6296778
197.714108
70.5096375
7.22623074
4.87687565
6.34248441
6.50452997
9.64769347
2.21247599
7.80117203
22.9843796
136.645771
7.4144886
2.53645752
16.2670372
14.3745938
41.2083073
2.56211031
14.2711837
23.4857617
8.92829899
23.8563299
23.7873803
2.91538399
5.65701688
24.9793767
1.49902726
4.72715548
15.656408
5.53521376
48.155449

140.488838
6.56594866
11.692985
15.9573799
19.9548685
82.7237402
104.411146
7.9249766
27.6759299
10.4152912
3.785168
78.5795122
3.24502108
0.55522771
3.58128447
7.03757384
56.1402726
4.52323916
12.2472588
40.9847066
5.46937939
3.89660377
28.5496131
9.18272834
5.07222846
2.8839175
0.89608862
5.54110732
2.42119399
8.59583565
56.708026
4.1711945
7.32558698
68.8024015
2.00299129
28.3967553
73.3707701
25.2421076
18.9870347
3.50470236
4.26722919
26.1402366
185.262831

28.8516582
47.5721656
3.82874569
54.8897218
11.7139814
5.7146638
1.89201992
24.7523903
1.90315529
6.1451905
3.6245018
15.4960584
7.04016421
32.633538
2.19318285
32.1900657
10.0978855
8.31167396
14.6030211
1.73281459
12.9407464
193.29509
6.81644204
33.2198436
14.1980733
107.04198
153.905004
120.892919
22.5560243
23.2182283
242.117374
4.73517357
2.68507672
67.7849583
1.96348098
79.6480659
178.457519
4.51432047
6.80926835
48.8700273
0.11908344
13.6709016
3.07335151

118.702574
14.0968538
1.6544863
10.6295991
3.15084462
14.8268182
27.9298544
12.2398272
8.68696637
13.0145823
3.23055777
6.56551334
4.28081149
2.83335951
102.619515
8.84959569
31.1072262
3.39015772
2.83881412
0.94225448
37.8425288
9.51155504
3.56443016
5.71407257
0.77822102
2.17660865
20.2504886
3.74997933
3.56419771
51.1115842
0.55687732
29.4185621
5.92717468
12.7102832
5.11833802
7.06537334
7.95292253
1.06565889
8.15254933
8.97404613
14.232061
0.09510114
21.0150704

7.02135825
30.6137854
23.9588944
12.0542946
33.8383219
64.9772988
19.8135312
16.2902866
59.405689
13.932974
14.4075299
28.3548025
1.98501058
36.7724331
167.630943
32.1687009
20.4056678
187.852927
0.98446676
16.070234
17.4620118
1.10858283
10.2804655
9.6149041
69.5134341
60.8723501
19.9595475
1.57242841
7.85941175
7.82279282
4.76445104
20.3095833
9.33628878
4.98172669
27.8458723
107.843342
5.9028194
22.7506335
87.9223275
21.0309353
6.17070233
75.8220899
2.4964372

9.39619667
173.1333
1.02386853
75.8877789
12.9472858
20.0566018
13.9672554
31.6775337
75.0107693
8.10750144
6.64463101
2.10227536
1.27816111
13.3387313
1.45301174
64.5363204
11.916416
8.41200178
4.66657241
1.59193291
